# Understanding cell‐cell communication and signaling in the colorectal cancer microenvironment

**DOI:** 10.1002/ctm2.308

**Published:** 2021-02-07

**Authors:** Shaikha AlMusawi, Mehreen Ahmed, Abdolrahman S. Nateri

**Affiliations:** ^1^ Cancer Genetics & Stem Cell Group, BioDiscovery Institute, Division of Cancer & Stem Cells, School of Medicine University of Nottingham Nottingham UK; ^2^ Department of Laboratory Medicine, Division of Translational Cancer Research Lund University Lund Sweden

**Keywords:** CellPhoneDB, patient‐derived explant (PDE), CITE‐seq, Colorectal cancer (CRC), CyTOF, patient‐derived organoid (PDO), patient‐derived xenografts (PDX), scRNA‐seq, Tumor microenvironments (TME)

## Abstract

Carcinomas are complex heterocellular systems containing epithelial cancer cells, stromal fibroblasts, and multiple immune cell‐types. Cell‐cell communication between these tumor microenvironments (TME) and cells drives cancer progression and influences response to existing therapies. In order to provide better treatments for patients, we must understand how various cell‐types collaborate within the TME to drive cancer and consider the multiple signals present between and within different cancer types. To investigate how tissues function, we need a model to measure both how signals are transferred between cells and how that information is processed within cells. The interplay of collaboration between different cell‐types requires cell‐cell communication. This article aims to review the current *in vitro* and *in vivo* mono‐cellular and multi‐cellular cultures models of colorectal cancer (CRC), and to explore how they can be used for single‐cell multi‐omics approaches for isolating multiple types of molecules from a single‐cell required for cell‐cell communication to distinguish cancer cells from normal cells. Integrating the existing single‐cell signaling measurements and models, and through understanding the cell identity and how different cell types communicate, will help predict drug sensitivities in tumor cells and between‐ and within‐patients responses.

## Graphical abstract

**Graphical Headlights**:

1. Multi‐cellular patient‐derived platform models intra‐/inter‐tumoral heterogeneity, and recapitulate human tumors’ complexity and microenvironment.

2. Simultaneous integration of multi‐omics data using multi‐cellular models enables identifying and comparing heterocellular cell‐cell communication in tumor vs normal tissues.

3. Multiple types of endpoint analysis using multi‐cellular patient‐derived models provide valuable insights for developing personalized cancer medicine, biomarkers and new therapies.

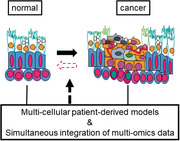

**ABBREVIATIONS**
CAFs: cancer‐associated fibroblasts
CITE‐Seq: cellular indexing of transcriptomes and epitopes by sequencing
CMS: consensus molecular subtypes
CyTOF: single‐cell time‐of‐flight mass cytometry
CRC: colorectal cancer
CRISPR: clustered regularly interspaced short palindromic repeats
DR‐seq: gDNA and mRNA sequencing
MALBAC: multiple annealing and looping based amplification cycles
ODEs: ordinary differential equations
PDE: patient‐derived explant
PDO: patient‐derived organoid
PDX: patient‐derived xenograft
RNA‐seq: RNA‐sequencing
scG&T‐seq: single‐cell genome & transcriptome sequencing
scM&T‐seq: single‐cell methylome and transcriptome sequencing
scTrio‐seq: single‐cell triple omics sequencing
scCOOL‐seq: single‐cell chromatin overall omic‐scale landscape sequencing
SCENIC: single‐cell regulatory network inference and clustering
TME: tumor microenvironments
WGA: whole genome amplification
WTA: whole transcriptome amplification

## 1 INTRODUCTION

Colorectal cancer (CRC) ranks as the third most common malignancy and the second leading cause of cancer‐related mortality worldwide.^1^ CRC is a heterogeneous disease, like other malignancies, making it a challenge for the optimization of treatment modalities in reducing morbidity and mortality.^2^ Typically, CRC initiation and progression occur as a result of sequential aggressive gene mutations and epigenetic alterations.^3^ The most frequently mutated genes in CRC comprise APC, 70%; TP53, 50%; K‐RAS, 40%; SMAD4, 25%; TGFβR2, 20%; FBXW7, 15–20%, and PIK3CA, 20%.^4^, ^5^, ^6^, ^7^ Mutation(s) of these genes and their respective signaling pathways result in major cellular consequences involved in apoptosis, proliferation, cell survival, and differentiation.^8^, ^9^ However, in recent years, multiple groups have generated large‐scale multi‐omics data profiles that have enabled the classifications of different cancers followed by comprehensive characterizations.^10^ This has gradually shifted the categorization of cancers from “mutation‐centered” toward a more “transcriptome‐based” molecular subtyping.^2^ Comprehensive genomic analyses have demonstrated that individual CRCs are unique, with a median of 76 non‐silent mutations each.^11^ To resolve the inconsistencies among the reported gene expression‐based classifications and to correlate CRC phenotype with clinical behavior, the CRC Subtyping Consortium unified six independent molecular classification systems and introduced a single consensus system known as the Consensus Molecular Subtypes (CMS).^12^ CMS has four distinct groups that enable the categorization of most tumors into one of four subtypes (Table 1).^13^, ^14^, ^15^ Even though CMS represents the current best description of heterogeneity at the gene‐expression level, it also correlates the epigenomic, transcriptomic, microenvironmental, genetic, prognostic, and clinical characteristics of CRC (Table 1).^10^, ^11^, ^13^

**TABLE 1:** Consensus Molecular Subtypes (CMS) of Colorectal Cancer. A single CRC classification system introduced by The CRC Subtyping Consortium that unified multiple independent molecular classification systems. MSI; microsatellite instability, CIN; chromosomal instability, DDR; DNA damage reaction

	CMS1 (MSI phenotype)	CMS2 (Canonical phenotype)	CMS3 (Metabolic phenotype)	CMS4 (Mesenchymal phenotype)	References
**Location; incidence**	Proximal; 14%	Distal; 37%	Mixed; 13%	Distal; 23%	^10^, ^11^, ^13^
**Characteristics**	Hyper mutated, microsatellite unstable, strong immune activation	Epithelial, marked WNT and MYC activation	Epithelial, evident metabolic dysregulation	Marked TGF–β activation, stromal invasion and angiogenesis.	^10^, ^11^, ^13^
**Associated mutations**	MSH6, RNF43, ATM, TGFBR2, BRAF, PTEN	APC, KRAS, TP53, PIK3CA	APC, KRAS, TP53, PIK3CA	APC, KRAS, TP53, PIK3CA	^10^, ^11^, ^13^
**Genomic Associations**	MSI, high mutation	CIN, low‐moderate mutation	CIN, moderate mutation	CIN, low mutation	^10^, ^11^, ^13^
**Epigenomic Associations**	High methylation	Low methylation	Moderate methylation	Low methylation	^10^, ^11^, ^13^
**Transcriptomic Pathways**	Immune activation, JAK‐STAT activation, Caspases	WNT targets, EGFR, VEFG/VEGFR, TGFB activation, cyclin upregulation	DDR, Glutaminolysis, lipidogenesis	Mesenchymal activation, immunosuppression	^10^, ^11^, ^13^
**Stroma‐Immune Micro‐environment**	Highly immunogenic, large immune infiltrate	Poorly immunogenic, innate immune response	Highly immunogenic	Innate immune response, EMT activation	^10^, ^11^, ^13^

## 2 THE CELLULAR CROSSTALK IN THE TUMOR MICROENVIRONMENT

**FIGURE 1:**
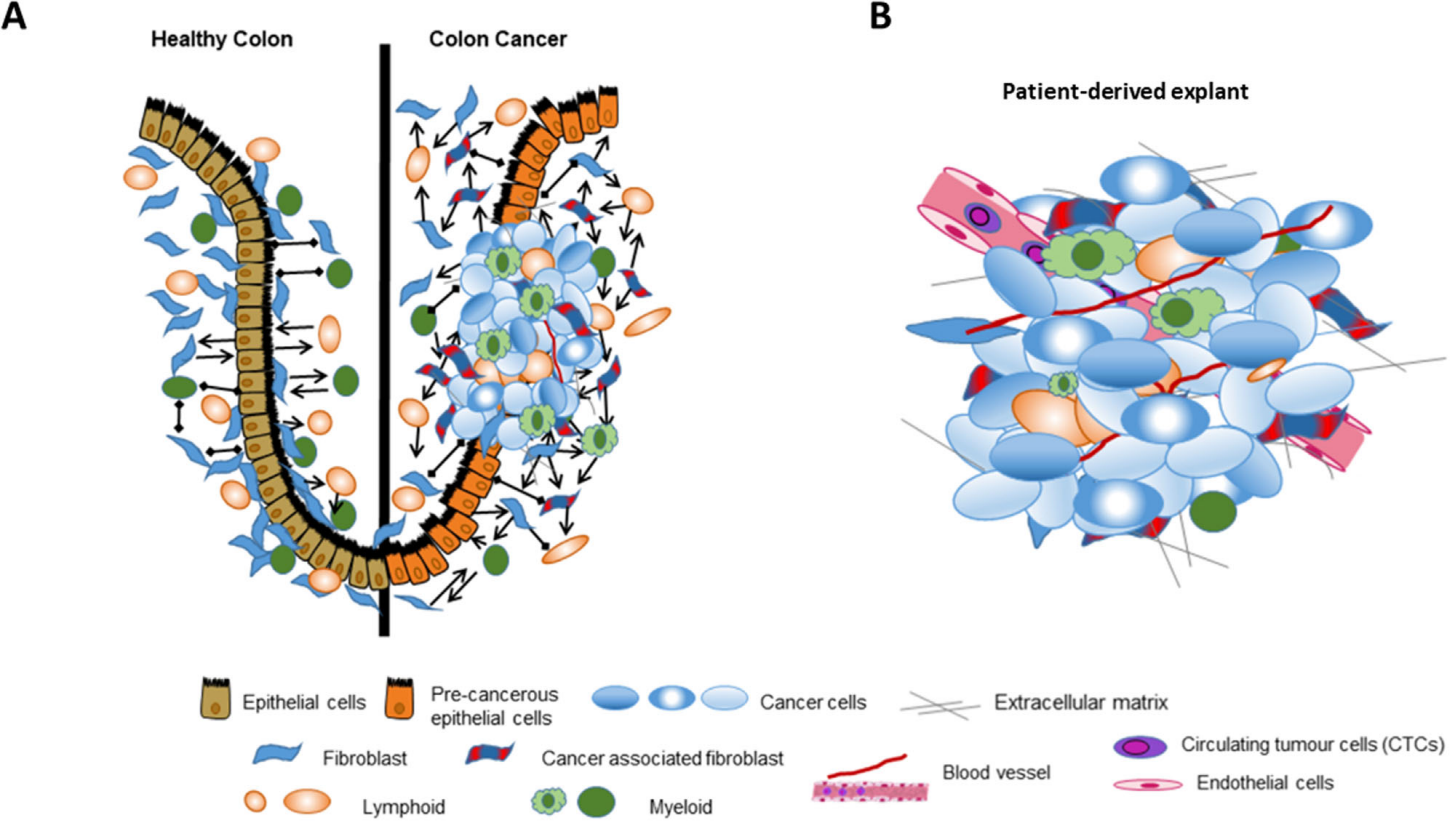
(A) Schematic representation of multiple cell‐cell communication in the colonic epithelium and colon adenocarcinoma. The arrows represent activated interactions between heterotypic cells. Left) “Normal” heterotypic cell‐cell interactions that maintain homeostasis and functionality of colonic epithelium. Right) The emergence of malignant phenotypes by oncogenic mutations, influenced by increased cell‐cell communication by different signaling pathways that were not activated before as well as the recruitment of more cell types. (B) Cellular heterogeneity maintained in a patient‐derived explant platform. In the patient‐derived explant system, the tumor samples are directly obtained from patients’ tissues following surgery as compared to other *in vitro* and *in vivo* approaches previously mentioned. Patient‐derived explant system provides an accessible model to study multiple cell‐cell communication interactions, and offer a promising platform for precision medicine approaches.

Within intestinal tissue, several different cell types collaborate through established interactions to form a functional organ utilizing a heterocellular system (Figure 1).^16^, ^17^ As CRC results from the deregulation and disruption of several signaling pathways tightly maintaining tissue homeostasis, understanding their dynamics is important to study the factors underlying CRC. Homotypic and heterotypic interactions between cells are therefore crucial for the maintenance of physiological functions changes in cell‐to‐cell communication and can induce tumorigenesis through different pathways.^18^, ^19^ To date, there is a large gap between our detailed knowledge of sub cellular processes and specific interactions that support tumor progression through heterocellular signaling at the tissue level. Instead, the dynamic analyses of cell‐cell communications and organogenesis have relied on model systems such as *Caenorhabditis elegans*, *Drosophila melanogaster*, *Xenopus laevis*, and zebrafish providing genetic mutations and reporter transgenic lines in cell competition manner involved in various physiological and pathological disease systems.^20^, ^21^ Cell competition typically originates from specific interactions between two cell types and is an interactive process wherein cells compete for certain fitness within a tissue environment. In zebrafish models, both its envelope layer and mesenchymal tissues with hyper‐activated Wnt/β‐catenin cells activate caspase‐3 and induce apoptosis.^20^ Typically, morphogen signaling forms an activity gradient in a signal‐dependent manner, whereas zebrafish model shows that unfit cells with abnormal Wnt/β‐catenin activity produce noise in the gradient. Communication between unfit and neighboring fit cells via cadherin proteins stimulates reactive oxygen species (ROS)‐mediated apoptosis of the unfit cells.^22^ In this manner, embryonic tissues eliminate excess noise and support proper formation and embryonic patterns. This system, however, is also relevant in aspect of CRC, as the function in the intestinal crypt undergoes active cell turnover and forms active Wnt/β‐catenin‐gradient.^20^, ^23^

### 2.1 Molecular mechanisms mediate cell‐cell communication

Recent studies indicated specialized cell surface protein complexes form epithelial cell‐cell junctions are essential for epithelial cell polarity and tissue integrity.^24^, ^25^ Upon the initiation of epithelial‐to‐mesenchymal transition (EMT), these junctions are deconstructed, disrupting tight cell‐cell contacts while the junction proteins are relocalized and/or degraded.^24^ The adherens junctions perform a pivotal role in regulating the activity of the entire junctional complex that comprises tight junctions, adherens junctions, and desmosomes.^26^ Cadherins are the transmembrane component of the adherens junctions that mediate cell‐cell adhesion.^27^ E‐cadherin is typically expressed by normal epithelial while disruption of E‐cadherin activity correlates with the formation of metastatic tumors.^28^ Inhibition of E‐cadherin activity was shown to change normal epithelial cells into invasive cells.^29^ This is accompanied by an increase of N‐cadherin expression and is commonly referred to as cadherin switching.^27^

It has been reported that epithelial‐derived cancer cells and cancer‐associated fibroblasts (CAFs) communicate through mechanical interactions via heterophilic adherens junction involving E‐cadherin on the cancer cell membrane and N‐cadherin on the CAF membrane.^30^ Labernadie et al concluded that CAFs favor invasion of cancer cells by pulling them away from the tumor, while cancer cells enhance their spread by polarizing CAF migration away from the tumor.^30^ Numerous studies have shown cadherin switching to be associated with tumor progression by mediating intercellular interactions that promote survival and migration of cancer cells.^31^ By transfecting with N‐cadherin, a non‐metastatic breast cancer cell line was transformed to a metastatic cell line.^32^ It is also likely that tumor cells have an increased ability to interact with endothelial cells by sharing the expression of N‐cadherin and this interaction promotes metastasis by allowing tumor cells access to the vasculature.^27^ Understanding how N‐cadherin influences cell behavior will provide a method to specifically combat its role in tumor growth, invasion, and metastasis.

Figure 1 illustrates cell–cell communication networks in a healthy and cancerous colon. When comparing the components of the tumor microenvironment, the colon adenocarcinoma presents greater heterotypic complexity which causes increased activation of signaling pathways that were not present in normal, healthy colon. Substantial evidence indicates that tumor stroma supports mutated colonic epithelial cells impacting and/or even hastening colorectal carcinogenesis.^33^ As such, the tumor microenvironment represents a modified pathological entity that evolves throughout cancer progression by setting up cell‐cell communication networks.^17^, ^33^, ^34^, ^35^ Multi‐omics data integration provides a more comprehensive dissection of tumor heterogeneity and cell‐cell communication networks.^36^, ^37^ By elucidating regulatory mechanisms within each CRC subtype, novel targets can be identified and tailored treatment strategies can then be produced in a subtype‐specific manner.^38^ In order to investigate this *in vitro*, there is a critical need for experimental models to recapitulate the intrinsic complexity and heterogeneity of a tumor. The optimal model can then be exploited using single‐cell technologies to better interpret cellular heterogeneity on the genetic, epigenetic, transcriptomic, and proteomic levels making it feasible to model cell‐cell communication networks. The primary focus of this review is to compare advanced in vitro mono‐cellular and multicellular models, and the single‐cell multi‐omics approaches to identify signaling pathways regulating cell‐cell communication networks. We will introduce the ideal multicellular model and proposed single‐cell technology, and discuss their application in translational CRC research context. For the purpose of this review, mono‐cellular models will typically represent one major cell class, e.g., epithelium while multicellular models will present more than one cell type in culture, e.g., immune cells or mesenchymal.

## 3 INVESTIGATING CELL–CELL COMMUNICATION IN CANCER MODELS

Cell–cell communication is fundamental to various processes, such as cell fate decisions, proliferation, migration, and homeostasis. CRC is a complex disease that thrives in a heterogeneous and adaptive tumor microenvironment.^39^ Despite the current understanding of organ microstructure and stromal composition, the need for complex models incorporating heterocellular interactions remains crucial in cancer research, especially to delineate the key molecular pathways and causative relationships involved in the tumor microenvironment, tumor dissemination, and overall carcinogenesis. A key challenge in understanding cell communication is to delineate the signaling pathways involved in cancer regulation. A number of studies suggest that signaling pathways can regulate each other by triggering sequential signaling events in cells.^40^ For example, Wnt protein mediates the balance between differentiation and proliferation, particularly in the stem cell niche. A recent study on Drosophila has elucidated a mechanism where an acyl group is covalently attached to Wnt, mediated by transmembrane protein Porcupine to attenuate Wnt activity.^41^ However, in most cases, the integrative signaling describing the spatial and temporal interactions between pathways are yet to be determined.

### 3.1 Preclinical models and their translational relevance

The high failure rate of preclinical compounds in clinical trials demonstrates the limitations of existing preclinical models. Approximately 10% of compounds progress successfully through clinical development where anticancer drugs have the highest percentage among all disease types.^42^, ^43^ Many of these drugs fail during clinical trials, especially during their phase III, which is the most expensive phase in drug development. Such failure is principally due to the lack of predictive patient outcome in response to candidate drugs.

Currently, in drug discovery, the compound screening starts with 2‐dimensional (2D) cell culture‐based assays. Established cell lines have been useful in understanding cancer with the advantage of easy control and analysis of expression however, a monolayer culture suffers from several limitations.^44^ Predominantly the proliferating cells adhere and grow on a flat surface, allowing unlimited access to nutrients and growth factors unlike tumors *in vivo*.^45^ Therefore, such cultures do not reflect cellular heterogeneity of the primary tumor, stromal‐cell communication, and tissue‐specific architecture.^46^

In recent years, several *in vitro* and *in vivo* preclinical models have been developed, including, spheroids, colonospheres, organoids, patient‐derived tumor organoids (PDO), patient‐derived tumor xenografts (PDX), and patient‐derived explants (PDEs). These models present valuable tools, for not only understanding cell‐cell communication, drug response, and the underlying mechanisms of tumorigenesis but also in drug discovery and the aspect of personalized medicine.^42^, ^43^ In patient‐derived xenografts, a segment of tumor tissue is obtained following surgery. This segment is implanted and subsequently passaged into immunodeficient mice.^47^, ^48^ CRC represents a unique illustration of patient‐derived xenograft studies, and may provide as an additional opportunity to improve clinical decisions.^42^ Moreover, the humanized patient‐derived xenograft models are an excellent platform to investigate the interactions between the immune system and microbiome. Recent evidence suggests that the human gut microbiome greatly contributes to CRC progression through the procarcinogenic activities of specific pathogens and their metabolites.^49^, ^50^ Typically, the success rates of CRC patient‐derived xenograft model development ranges between 64–89% ^51^, ^52^. In CRC modeling, patient‐derived xenograft retains the authentic characteristics of the patient's tumor tissue, including histopathologic architecture, genomic signature, intratumoral clonal heterogeneity, chromosomal instability, and drug responsiveness.^51^, ^52^, ^53^ The key driver mutations, including KRAS and PIK3CA, remain consistent along the passages.^54^ In a recent study, Isella *et al* reported that the stromal transcripts derived from CRC patient‐derived xenografts successfully recapitulated the prognostic mesenchymal gene signature of human CRC tumors, indicating the reliability of CRC patient‐derived xenograft in modeling the reciprocal paracrine signaling between cancer cell and murine stromal cells.^55^ An important application of patient‐derived xenograft is personalized cancer treatment by utilizing heterogeneous patient tissue and the ability to model cancer as a whole.^18^ It has been reported that patient‐derived xenograft models represent the clinical response to therapy better than traditional xenografts.^48^, ^56^ As previously outlined in Table 1, CMS4 tumors are characterized by activation of pathways related to Epithelial‐Mesenchymal transition (EMT) and stemness, such as TGF‐β and integrins, and are mostly derived from stromal cell infiltration of adjacent cancer tissue. It has been shown that the use of TGF‐β inhibitors in CRC patient‐derived xenograft models had blocked the crosstalk between cancer cells and the microenvironment and therefore reduced metastases.^57^

However, the clinical applicability is limited due to time requirements, lack of penetration of delivery systems, high costs associated with patient‐derived xenograft systems, the large sample size, and the influence of infiltrating murine stromal cells on the tumor.^58^, ^59^ Consequently, the more times a patient‐derived xenograft tumor is passaged through mice, the more transcriptionally “mouse‐like” it becomes and over time, the human stromal cells are replaced by mouse stromal cells.^51^, ^60^, ^61^ The depletion of human stromal and immune cells is a major limitation of patient‐derived xenograft models for studies of tumor microenvironments and metastasis.^51^ Moreover, the loss of human CAFs, endothelial cells, and immune cells over time has been characterized as a pitfall of the patient‐derived xenograft model. Based on the limitations of using 2D culture systems and patient‐derived xenograft, significant effort has been put forward to develop three‐dimensional (3D) culture models that provide a more relevant and practical alternative to investigate the pathophysiology of human cancer. To model cell‐cell interaction *in vitro* both mono‐cellular and multicellular models each provide their own set of strengths and limitations. The following section provides a comparative evaluation of different models summarized in Table 2.

**TABLE 2:** **Advantages and disadvantages of current experimental models**. Comparative summary of mono‐cellular and multicellular culture models that have been highlighted. The advantages and disadvantages provide insight toward the relevance of each method to model cell‐cell interaction networks. The *ex vivo* patient‐derived explant platform illustrates an optimal model to study cell‐cell interactions by maintaining the heterogeneity of the original tumor

	Model	Advantages	Disadvantages	References
**Mono‐cellular**	***In vitro* 2D culture system**	CRC cell lines are easily expandable Amenable to genetic modification Simple, reproducible, low‐cost	Loss of tissue‐specific architecture Histological and genetic features are different to native tumors Lack cell diversity and cell‐cell and cell‐ECM interactions	^44^, ^92^, ^184^
	***In vitro* spheroid**	Retain characteristics of physiological structure and function of source tissue: Preserved genomic and transcriptomic characteristics Signaling established between cells Expanded long term *in vitro*	Unable to form tissue‐like structures Reproducibility is questionable due to self‐organization Separation of single‐cells takes several hours to a few days	^64^, ^102^, ^185^
	***In vitro* organoid**	Multiple differentiated cell types Signaling pathways governing organoid formation are identical to that during *in vivo* organ development and homeostasis Intercellular communication and organization networks are more successful than 2D culture systems.	Pure epithelium Scaffolds of natural origin are not chemically well defined Exchange of material through slow infiltration rather than blood vessels	^186^, ^187^, ^188^
**Multi‐cellular**	***In vivo* PDX model**	Established human immune response Partly recapitulates tumor microenvironment High predictive value	Do not retain original cell properties Cannot reproduce heterogeneity of human tissue Long time to establish and high cost Species‐specific differences	^47^, ^56^, ^189^
	**PDO co‐culture**	Models heterotypic interactions Retains the cellular composition of patient tumors Provides a reliable model for drug testing compared to mono‐cellular culture models	Variability in terms of composition and structure due to matrix Difficult to compare data generated from different laboratories due to variability Challenge to keep proliferative state	^18^, ^99^, ^190^
	***Ex vivo* PDE platform**	Patient‐relevant material Tumor retains proliferative Maintains cellular heterogeneity Allows correlation of drug responses with pathology and patient characteristics Rapid drug response data can be collected Relatively inexpensive	Only applicable to surgically resected tumors Short time frame Single‐cell multi omics approaches can cause disruption to samples Experimental results affected by tumor integrity	^111^, ^115^, ^116^

## 4 MONO‐CELLULAR MODELS

It is necessary to maintain or recreate the typical architecture of a tumor, to investigate cell‐cell interactions regulating tumor signaling pathways successfully. 3D *in vitro* models have been used as an intermediate model between *in vitro* cancer cell line cultures and *in vivo* tumors.^62^, ^63^ 3D models can produce *in vivo*‐like iterations and confer complexity.^21^ Typically, 3D mono‐cellular models include organoids and spheroids.^64^

### 4.1 Spherical models

Spheroids form as cell aggregates or spheres cultured primarily in suspension and are mostly enriched in stem‐like population.^63^, ^65^ The stem cell medium is devoid of fetal bovine serum (FBS) and is supplemented with factors that favor stem cell growth including basic fibroblast growth factor (FGF) and epidermal growth factor (EGF).^63^ Spheroids from primary colorectal cancer were first established from CD133+ colon cancer cells and were shown to reproduce the same histological features of the original tumor in immunocompromised mice by maintaining properties of self‐renewal.^66^, ^67^ Essentially, their spherical morphology decreases cell viability and forms hypoxic and necrotic cores that very closely recapitulate the conditions found in solid tumors, including CRC.^62^, ^68^ Various stages of cells comprise these 3D spheroids or aggregates, including proliferating, quiescent, apoptotic, hypoxic, and necrotic cells. This recapitulates the physiological characteristics of tumors with regard to cell‐cell contacts.^68^ Hence, they are more likely to provide the structural and functional tumor heterogeneity, cell‐cell and cell‐environment interactions, and overall cell function.^69^, ^70^

“Colonospheres” have since been used to investigate cancer stem cell (CSC)‐related characteristics, cell‐cell adhesion, chemo‐resistance, initiate xenograft tumors as well as tumorigenicity by single‐cell cloning.^71^, ^72^, ^73^ Additionally, colonospheres present a valuable tool for investigating the cell fate decision and cell‐cell interactions.^65^ Moreover, spheroids cultured from primary colorectal cancer cells have been shown to retain cell‐cell contact enabling evaluation of chemo‐sensitivity and signal pathway activation in individual patients.^74^ In addition to the tumor‐initiating capacity of this culture model, methods for generating spheroids are simple, cost‐effective, and highly reproducible. However, spheroids present a poor *in vitro* model for healthy epithelial tissues due their inability to form tissue‐like structures.^75^, ^76^

### 4.2 Organoid models

Organoids, on the other hand, originate from either pluripotent or adult stem cells that give rise to organ‐specific cell types. Although 3D organoids could mimic some of the *in vivo* epithelium features, they lack the niche consisting of stromal cells, immune cells, and vasculature and are unable to mimic the *in vivo* microenvironment.^44^ Organoids require a matrix to propagate and consequently, acquire a more ordered assembly than spheroids that more typically recapture complex tissue architecture.^75^ The first adult stem cell‐derived organoid cultures were established from Lgr5‐expressing mouse intestinal stem cells, where the culture condition successfully mimicked the intestinal stem cell niche.^77^, ^78^ Since then, organoid cultures have been established in a variety of tissues, including the colon.^47^ Organoids serve as excellent *in vitro* model to study tumor microenvironment, specific cell‐type response to drugs and enable cells to grow in a more similar manner to that of living organisms.^79^, ^80^, ^81^, ^82^ In a CRC organotypic model, extracellular vesicles from colon fibroblasts grown in hypoxic conditions showed an increase in neoplastic organoids, suggesting a role of fibroblast‐derived extracellular vesicles in tumorigenesis ^83^. An intestinal organoid consists of multiple epithelial cells.^77^, ^84^, ^85^ In 2011, human tumor organoids were first generated from the colon.^86^ It has also been reported that human colon tissue obtained from colonoscopy biopsy samples, surgical resections, or single EphB2+ stem‐like cells can be cultured as organoids.^86^

Typically, tumors *in vivo* are composed of proliferating neoplastic parenchymal cells and supportive stroma that constitutes half the mass of most malignant tumors.^87^ Importantly, the parenchymal cells determine the growth and differentiation of the tumor, while stroma contributes towards tumor progression. The spatial distribution of cancer and stromal cells within the tumor microenvironment can determine the cell‐cell interaction and can influence the proliferation, differentiation, morphology and a range of cellular functions.^88^, ^89^ While organoid technology presents the great advantage of studying epithelial tissue, organoids still do not fully recapitulate all the characteristics present *in vivo*. A major limitation is their 3D closed geometry which complicates access of the organoid‐analogue lumen in intestinal organoids for the use of conventional assay, high throughput screening, drug absorption, and delivery, and microbe‐epithelium interactions.^90^ Moreover, the invasive procedures to obtain the intestinal and colonic patient biopsy samples present a major challenge for larger‐scale culture of human intestinal organoids.^91^ Although 3D colonospheres and organoids both provide an *in vitro* multicellular model, heterotypic cell‐cell interactions are not present. Consequently, studying the interactions between carcinoma and intratumoral stromal cells is not possible within a mono‐cellular culture model. Therefore, to evaluate cell‐cell communication in CRC, model systems must recapitulate cellular heterogeneity in which the diverse microenvironment is present. An ideal 3D culture model would stimulate tissue‐specific physiology where cells can proliferate, aggregate, and differentiate, and include cell‐cell and cell‐extracellular matrix (ECM) interactions.^92^ The current limitation of organotypic cultures lacking the multicellular representation of the tumor microenvironment has been partly overcome by the ability to retain immune cells with air‐liquid interface (ALI) patient‐derived organoids, as well as, by co‐culturing the patient‐derived organoids with a variety of cell types, including patient‐derived immune cells or cancer‐associated fibroblasts.^18^, ^93^, ^94^, ^95^, ^96^ These 3D patient‐derived organoids systems retain autologous immune cells enabling immunotherapy studies and thereby offer as promising tools to model heterotypic interactions between cells that compose the tumor microenvironment. Table 2 provides a comparative evaluation of different CRC models.

## 5 MULTICELLULAR MODELS

As yet, the ability to predict patient tumor response to cancer therapy remains a major challenge. It has been repeatedly reported that tumor microenvironment and heterogeneity can limit the predictive response of existing biomarker‐based therapeutic strategies.^88^, ^89^ A major challenge in the development of relevant CRC models remains to be the lack of heterotypic cell interactions. Recent advances in organoid and spheroid models allow co‐culture with heterotypic cells enabling the formation of a heterocellular system that can reproduce cell‐type‐specific signaling networks.

### 5.1 3D co‐culture systems: CRC organoids and CRC spheroids

A recent study demonstrated that co‐culture of CRC organoids with high mutational burden and autologous peripheral blood mononuclear cells (PBMCs) function provided antigen‐specific stimulation of T cells in the PBMC fraction.^95^, ^97^ Moreover, cytotoxic killing was higher in CRC organoids co‐cultured with tumor‐infiltrating lymphocytes generated from patients with a complete response to therapy, than that of the ones derived from therapy nonresponders.^97^, ^98^ Therefore, such co‐culture assays may provide new insights in cancer immunotherapy in future. A study by Qin *et al* focused on intestinal organoids co‐cultured with fibroblasts and macrophages.^99^ By analyzing single‐cell posttranslational modification signaling in co‐cultured organoids, their method revealed cell‐type specific signaling networks that were hidden in organoid monocultures. Regardless, previously reported CRC 3D co‐culture systems have enabled the study of metastasis and interactions of immune cells and fibroblasts. The tumor‐stroma co‐cultures consisting of ECM fibers and micro‐architecture, induced an epithelial phenotype in CRC cells.^100^ Recently, a 3D model based on CRC multicellular tumor spheroids was developed by combining epithelial colon cancer cells, intestinal fibroblasts, and monocytes. This model successfully mimicked tumor characteristics as cells underwent spatial organization and produced extracellular matrix, thereby presenting a valuable model to investigate nano‐therapeutic strategies in CRC.^101^ Another study that focused on the co‐culture of tumor‐derived spheroids with immune cells revealed the ability to assess infiltration, activation, and function of T and natural killer (NK) cells toward human colorectal tumors.^102^ It was reported that resistance mechanisms are used by tumor cells to evade immune recognition by HLA‐E upregulation. Although the NKG2A‐HLA‐E pathways has been described previously,^103^ their model, in line with other co‐culture 3D models, demonstrates a heterocellular cell culture system that has a viable tumor microenvironment with functioning intercellular communication. Moreover, Hoffman *et al*. generated spheroids from tumor cell lines solely, tumor cell lines co‐cultured with PBMCs and spheroids directly prepared from colon cancer tissues.^104^ It was demonstrated that with the addition of PBMCs the co‐cultured spheroid responded differently to 5‐FU/Oxaliplatin treatment compared to the homotypic spheroid and had increased resistance. More importantly, the colon cancer tissue spheroid was reported to have three distinct response patterns that were not detectable in the 3D cell line models. This highlights the importance of retaining the cellular composition of patient tumors for reliable modeling. The complexity, heterogeneity, plasticity, and diversity of the human tumor microenvironment leaves models with inaccurate deductions regarding clinical responses. Although several interesting observations suggest that 3D co‐cultures are more relevant, these models are expensive, highly variable, and are therefore not suitable for large‐scale screening.^101^

### 5.2 3D co‐culture systems: patient‐derived organoids

Although patient‐derived organoids (PDO) developed from primary or metastatic tumor tissue can be expanded for molecular profiling, PDO models are inherently limited in their ability to reflect the tumor microenvironment *in vitro* as they comprise exclusively of epithelial cells.^105^ The lack of stromal cells; such as CAFs, immune cells, vasculature, etc. (Table 2), in patient‐derived organoid models poses a major problem as these tumor microenvironment components play important roles in cancer development and progression, from the regulation of cancer cell proliferation and stem cell maintenance to drug resistance and prognosis.^55^, ^57^, ^106^, ^107^ However, the success rate of establishing organoids from untreated primary CRC patients is about 90%, and 70% when using biopsy of metastasis CRC. In addition, it takes about 30–80 days to establish a frozen biobank of 1 × 10^6^ CRC organoids.^108^ Therefore, the timing and cost‐effectiveness of organoid‐based approach is not often feasible. Having provided an important tool in translational research, patient‐derived organoids also limit our understanding of the stroma/immune signaling influence on tumor maintenance and drug response. These deficiencies intensely underscore the need for novel tools to precisely match patients with effective therapies.

### 5.3 Patient‐derived explants

**FIGURE 2:**
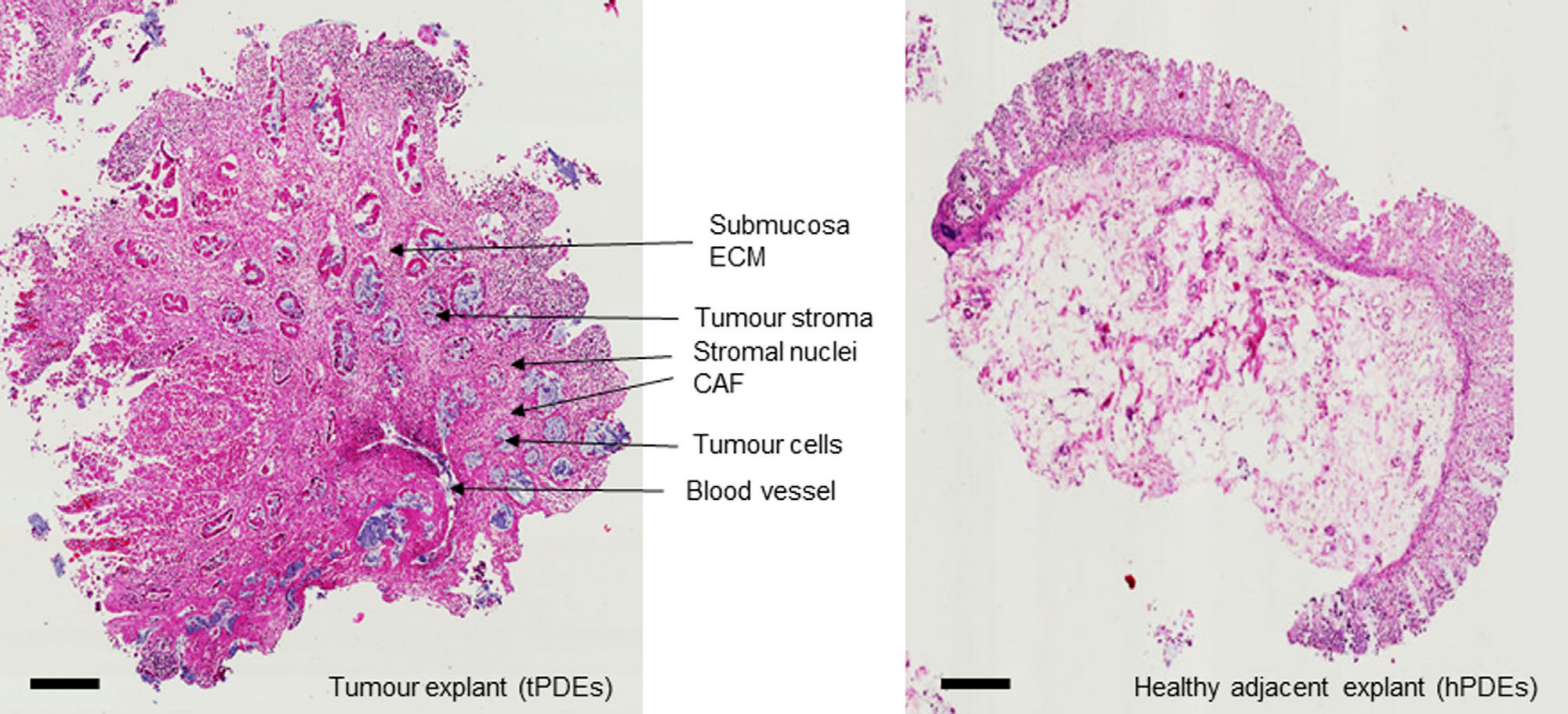
Histological examination of a colorectal cancer‐patient‐derived explant model. Hematoxylin and eosin (H&E) staining, scale bar: 100 μm. Tissue structures have been identified and labeled. Maintenance of original tissue organization, architecture, and cellular heterogeneity is observed.^110^

In recent years, *ex vivo* patient‐derived tissue explant cultures of human tumors have demonstrated that they can reliably manifest the tumor growth *in vivo* maintaining the original tissue organization and architecture (Figure 2).^109^, ^110^ This platform is applicable to multiple solid tumor types, including prostate, ovary, endometrium, renal, sarcoma.^111^ Others and we have recently developed and validated a short‐term patient‐derived explant culture of human colon cancers for the functional assessment of primary colorectal carcinoma, drug discovery, and personalized drug screening.^88^, ^110^, ^112^

The patient‐derived explant model involves the *ex vivo* culture of freshly resected human tumor tissues using protocols ranging from total immersion of tissue sections in medium, on the grid, or in gelatin sponge scaffolds.^113^, ^114^ Though patient‐derived explants have been reported to be more viable in short‐term culture, they can also grow at high frequency for a long period and still present many of their *in vivo* properties.^88^, ^109^ Such properties include the native 3D tissue architecture, preservation of the spatial distribution of tumor and stromal cells, tumor microenvironment, cell viability, key oncogenic drivers, retention of differentiated function, and the three‐dimensional growth of different types of cells from a single tumor (Figure 2).^111^, ^115^, ^116^ In cancer modeling, the conventional model systems fail to recapitulate the tumor microenvironment, which often leads to poor correlation with clinical outcomes (Table 2).^117^ Patient‐derived explants present a model that can preserve heterogeneity, capture the microenvironment, and therefore have useful applications for translational cancer research, including CRC. Previously, tumor explants obtained from CRC patients have been used to investigate whether the microenvironment of earlier staged tumors is as suppressive as that of the later stages.^118^ Moreover, the monocyte‐derived dendritic cell (MDDCs) of non‐metastatic CRC patients were reported to secrete low levels of IL‐12p70 in response to lipopolysaccharides. Additionally, it has been established that the tumor microenvironment can inhibit dendritic cell maturation in CRC using the tissue explant model.^118^, ^119^

Additionally, the patient‐derived explant platform provides a unique patient‐relevant model system for the preclinical evaluation of novel anticancer agents. When combined with tumor stratification approaches, this platform provides a direct evaluation of drug responses on an individual patient's tumor, which can be further amended by contemporary genomic analysis. Patient‐derived explants exhibit high potential in personalized drug treatment by permitting drug efficacy evaluation from individual patient material. Patient‐derived explants may provide an excellent model to improve our understanding of cell‐cell communication, tumor heterogeneity within individual patients and between different patients, and response to treatment. A study on CRC tumor explants revealed a pro‐tumorigenic mechanism in which the components of the immune system that are exploited in metastases were found to ultimately promote tumor growth and invasion.^120^ However, this mechanism can be targeted by blocking CCR5, which then causes antitumoral repolarization of macrophages and mitigation of the pro‐tumor inflammatory microenvironment.^120^

Recently it has been established that patient‐derived explants can be manipulated using hormones, siRNA, or drugs and their responses can be assessed using an array of robust quantitative evaluation of clinically relevant endpoints techniques, including immunohistochemistry, real‐time qRT‐PCR, and genome‐wide molecular analyses.^111^, ^121^ However, endpoint evaluation of drug responses may also include disintegration of the explants for subsequent growth assays that use the enzymatic digestion of tumors or RNA/DNA/protein/metabolite analysis. Alternatively, whole explants can be processed for formalin fixation and paraffin embedding for spatial profiling of key biomarkers using immunostaining techniques.^114^, ^117^ Although, the *ex vivo* patient‐derived explant platform has many advantages over other 3D pre‐clinical models,^117^ whether such platform can be routinely employed as model systems in the evaluation of new therapeutics is yet to be determined. Moreover, it has also been demonstrated that the establishment of a living biobank of tumor organoids may facilitate the integration of genomic data with drug screening, but this platform may not be feasible to utilize for all cancer patients.^122^

## 6 BUILDING CELL–CELL COMMUNICATION FROM OMIC TECHNOLOGIES

Communication is mediated by specific ligand‐receptor interactions. Identifying the set of “sender‐receiver cell pairs” will enable the characterization of entire networks in malignant tissue that involve the participation of both neoplastic and stromal cells. As mentioned, patient‐derived explants provide the optimal model to identify cell–cell communication networks by providing an intact heterocellular system (Figure 2). The ability to simulate interactions between cells of the tumor microenvironment *in vitro* assists current knowledge on cancer growth and helps to identify novel drug targets. The recent development of high‐throughput single‐cell omics technologies brings exciting possibilities to study signaling pathways that are regulated between ligand‐receptor networks.^123^ Such findings can be used to understand the molecular cascades involved in tumor initiation and progression, elucidate the functional relationships of biomolecules within individual cells or cell types, and between populations. Additionally, combining research based on single‐cell sequencing and the established CMS categories would offer the collective opportunity to elucidate a more refined subtype‐specific CRC cell origin and help characterize the different paths of evolution.^2^

It is important to note that the majority of colorectal cancer network methodologies and applications have depended on omics data derived from bulk tissues, primarily focusing on predicting gene regulatory networks within and between tissues. However, bulk tissue networks mainly represent the cell population's average activities and, thus, cannot capture cells’ individual behavior but rather those involved in similar pathways or functions.^124^, ^125^ Ultimately, this bulk analysis provides a superficial insight towards direct interaction networks. Even so, this data can be supported with single‐cell multi‐omics approaches to infer directionality and facilitate interpretability. Furthermore, the interpreted cell‐cell communication networks based on tissue samples cannot confirm the specific molecules that participate in cell communication. Yet, these findings provide a base to further define these speculative interactions and confirm the molecules involved in tumor‐associated biological functions.

## 7 MULTI‐OMICS APPROACHES IN COLORECTAL CANCER

Omics approaches can help to identify driving factors and causal relationships within colorectal cancer. Recently, Ayiomamitis *et al* evaluated the differences in COX‐2 expression between epithelial and stromal cells of the tumor and adjacent normal tissues using biopsied tissue from CRC patients.^126^ In line with previous studies, their research demonstrated that COX‐2 expression is mainly stromal in the adjacent normal tissue and is directly implicated in angiogenesis, by favoring the survival of abnormal cells. This allows local growth of the malignant tumor and progression within the normal tissue.^127^ Activated fibroblasts surrounding tumors also participate by constructing a scaffold for the tumor to metastasize.^128^ At diagnosis, more than 50% of CRC will develop metastatic disease.^129^ A proteomic study based on 10 patients compared non‐metastatic and metastatic primary CRC tissues against their normal tissues.^130^ Their research presented upregulated and downregulated differentially expressed proteins (DEP) unique to non‐metastatic or metastatic CRC, or shared by both. The greatest number of unique DEPs were associated with metastatic tumors. Furthermore, enrichment analysis was used to identify 962 specific pathways associated with metastasis. However, as mentioned by the authors, further research on signaling pathways and signaling molecules is required. Hence, the following sections provide an extensive explanation of the different single‐cell omic technologies applicable in deciphering cell‐cell communication within the CRC tumor microenvironment which have yet to be carried out.

It is accepted that CRC cells activate fibroblasts into cancer‐associated fibroblasts and in return, the cancer‐associated fibroblasts’ secretome promotes cell proliferation and metastasis of the tumor. Although the root cause of cancer is usually genetic or epigenetic alterations, the progression of cancer is associated with intricate crosstalk between tumor cells, surrounding stromal cells and the ECM.^131^ Table 3 provides examples of released factors by different cell types of the colorectal‐cancer tumor microenvironment as well as their stimuli. Several systematic approaches have been proposed for selecting important biomarker candidates. However, single‐gene expression analysis may not provide an informative conclusion, while a combination of omics datasets would provide a broader perspective. For example, increasing evidence has suggested that left‐sided colon cancer and right‐sided colon cancer have distinct clinical characteristics and can potentially be treated as two different diseases.^132^ A multi‐omics study characterizing the somatic mutations, genome‐wide transcriptional (mRNA and miRNA) and epigenetic profiles of left‐sided and right‐sided CRC was carried out by Hu and co‐workers.^133^ By the integrated comparison of mutations and mRNA expression alteration it was found that the PI3K signaling pathways were more prevalent in the right side and frequently exhibited cross‐talk with the RAS and p53 pathways compared to the left side. These findings provide insight into the molecular mechanisms involved in cell‐cell communication that can be targeted when treating the two different sides of the same disease.

**TABLE 3:** Cellular components of the colorectal cancer microenvironment. The following table lists several cell components of CRC TME along with a description of their role in tumor suppression or tumor progression. The main stimuli the cells respond to and the factors the cells release are also noted

Cell type	Role in CRCStimuliReleased factors	References
**Cancer‐associated fibroblasts**	Promote tumorigenesis, migration and supports survival of malignancy PDGFα/β, TGFβ, IL‐4/6, IGF‐II, FGF2, ROS, PGE, VEGF, EMMPrin MMPs, VEGF, PDGF, IL‐1/6/8, OPN, HGF, IGF1, IGF2, EGF, MIF, FGF7, PGE‐2, FGF2, CXCL‐12, VTN‐N, miR‐200b, miR‐155, OPN, TGFβ	^19^, ^191^, ^192^
**Myeloid cells**		
**Mast cells**	High mast cell density has been related to tumor aggressiveness and reduced survival SCF, NGF, C3a, IgE, C5a, IgG, IL4/5, TLR ligands, IFN‐γ VEGF, FGF2, CCL‐2, Heparin, Histamine, TNFα, GM‐CSF, ANG‐1, tryptase, IL‐3/5/6/8/10/13	^193^, ^194^
**Neutrophils**	Neutrophils induce tumor angiogenesis and correlated with poorer clinical outcomes in patients diagnosed with advanced CRC. TME is able to polarize neutrophils into cells with an N1 or N2 phenotype. N1 phenotype has anti‐tumor effects and N2 has pro tumor effects. N1: IFNβ, C5a, CCL3, IL‐2/5/6/8/12/16 TNFα, ROS, CTS‐G, ELA1, CCL3, GM‐CSF, IL‐1β/9/10/12 N2: IL‐1/2/5/6/8/12/16, C5a, PGE2, CCL3 ARG, MMP8, ROS, OSM, TGFβ, HGF, IL‐6/8/10/17, VEGF, PDGF, TNFα	^195^, ^196^
**Lymphoid cells**		
**CD8+ T cells**	Part of the adaptive immune response. The density and location of these cells within CRC samples is a positive indicator of patient survival APC antigens, IL‐1/2/6/12, IFN‐ α/β, histamine IFN‐γ, TNFα, GRZ‐B, PRF1	^197^
**Natural Killer cells**	NK cells have a limited capacity to infiltrate the CRC microenvironment. NK and CD8+ T cells infiltration of the CRC microenvironment is reported to correlate with favorable prognosis. PRF1, TGFβ, kynurenines, NO, IL‐2/10/12/15/18 Pro‐inflammatory cytokines, CRZ‐B, PRF1, IFN‐γ	^198^, ^199^
**Other cells**		
**Endothelial cells**	Key role in the development and function of blood and lymph vessels. High vascular density at the CRC invasion front is reported to be directly associated with tumor recurrence, metastasis and patient mortality. PIGF, VEGF, PDGF, HIF‐1/2α, IL‐8, FGF, ANG1/2, TGFβ VEGF, PDGFβ, HB‐EGF	^200^
**Pericytes**	Support blood vessel formation and function. Established reciprocal communications with endothelial cells. PIGF, VEGF, PDGF, ANG1/2, HIF‐1/2A, MMPs VEGF, ANG1/2	^201^, ^202^

## 8 APPLICATION OF SINGLE‐CELL MULTI‐OMICS FOR CRC CELL COMMUNICATION

More recently, Yi *et al* utilized colon cancer cell line (SW480) to investigate whether intratumoral Wnt heterogeneity could directly drive EMT heterogeneity using scRNA‐seq. Assay for Transposase‐Accessible Chromatin with high‐throughput sequencing (ATAC‐seq) was used and identified a new EMT‐promoting transcription factor (TF) RUNX2. Knockdown of *RUNX2* inhibited migration and metastasis of cells which exhibited high Wnt activity. Whereas, when overexpressed with RUNX2, cells with low Wnt activity were promoted to migrate. Clinical evidence shows that RUNX2 expression is positively associated with metastasis progression and lower survival for CRC patients. It has been previously reported to induce bone‐mimetic gene expression pattern promoting metastasis of breast and prostate cancer, and to enhance expression of EMT‐associated genes in prostate, thyroid, and breast cancers. However, this study did not identify common metastasis‐related targets, suggesting that crosstalk between EMT‐TFs in this study is distinct to prostate, thyroid and breast cancers. Thus, it is important to identify the TFs regulatory partners especially through a model that presents heterogeneity such as patient‐derived explants.

It is important to note that only three studies have compared the tumor microenvironment of endoscopic biopsies and surgical specimens in CRC.^134^, ^135^, ^136^ All three reports demonstrated a weak correlation between the biopsy tumor microenvironment with the tumor microenvironment present in the total resection specimen. As further studies are needed to focus on the clinical relevance of singular patient‐derived explants (biopsies), it would help to map the tumor microenvironment by using multiple samples throughout the tumor, in addition to sampling before and after treatment to evaluate definitive effects of the therapy. The previous sections in this review which describe single‐cell multi‐omics approaches can potentially be employed to compare multiple sections of the tumor microenvironment and provide a comprehensive background on the clinical relevance of patient‐derived explants. Additionally, bioinformatics tools and computational resources can be used for measuring cell‐cell communication (Table 4). By utilizing the state‐of‐the‐art omic technologies, the data gathered would provide insight towards the prospective of using patient‐derived explants as a preclinical model to recapture the endogenous cell‐cell communication networks. Importantly, this would elucidate the cellular functions within the tumor microenvironment while considering the community context of each cell. Unlike other preclinical models, heterogeneity is maintained in patient‐derived explant models. Therefore, studies based on the protein messages passed between cells as well as, the expressed messenger molecules and their associated pathways, will be relevant to the specific patient from which the biopsy was obtained. This provides insight towards an already sustained and coordinated multicellular tissue as opposed to other models that favor simulations.

**TABLE 4:** Existing bioinformatics tools for modeling cell‐cell communication

Tool	Method overview	Advantages	Disadvantages	References
**SCENIC**	Transcription factor (TF) target‐based regulation. Combines TF regulatory relations (GENIE3) with TF‐binding motif analysis.	Robust against dropouts, get a TF score for individual cells	Limited to TF‐based relations	^176^
**SCODE**	TF expression dynamics (pseudo‐time) and TF regulatory relations (GENEI3)	Relational expression using linear regression, fast algorithm	Dimension reduction necessary for computing speed, assumes all cells on the same trajectory	^177^
**PIDC**	Uses partial information decomposition to find dependencies in the expression patterns of genes	Compared to correlation, more gene dependencies are identified	Influences by data discretization, method developed for sc‐qPCR	^179^, ^203^
**SoptSC**	Estimate interaction between two cells based on expression of ligand, receptor and downstream pathway target genes. Output gives up‐ and down‐regulated interactions	Incorporates target genes of pathways and directionality	Requires curation of ligand‐receptor interactions and downstream pathways	^174^, ^176^
**scTensor**	Tucker decomposition with ligand‐receptor interactions as hypergraphs. Many options for interaction, expression and pattern visualization.	Ligand‐rector pairs across multiple cell types – more reflective of biology	Requires curation of ligand‐receptor interactions and creates average of the single‐cells to cell type level.	^204^
**iTALK**	Enumerates differentially expressed ligand and receptor values. Produces up‐and down‐regulated interactions	Directionality of interaction can be inferred	Requires curation of ligand‐receptor interactions. Can not reveal novel interactions.	^204^

## 9 CHALLENGES AND PERSPECTIVES

Unfortunately, the current gold standard *in vitro* and *in vivo* preclinical approaches are all limited by their inability to capture the full biological approximation of the native tumor, resulting in poor mapping to clinical outcomes. Even so, colorectal cancer‐patient‐derived explant models critical physiologic parameters, present complex multicellular architecture, barriers to mass transport, and extracellular matrix deposition. However, before we can routinely employ colorectal cancer patient‐derived explants to model therapy response, further characterization of cellular and molecular properties and methodologic framework that maximizes their clinical and translational applications are required. Technological challenges also arise as the composition of cellular models remains variable even when they contain the same cell types, cell number, and transcriptional states of individual cell types. Therefore, we provided an overview of how the new single‐cell omics technologies can allow the study of cell‐to‐cell communication within a colorectal cancer patient‐derived explant.

### 9.1 A single‐cell multi‐omics approach

**FIGURE 3:**
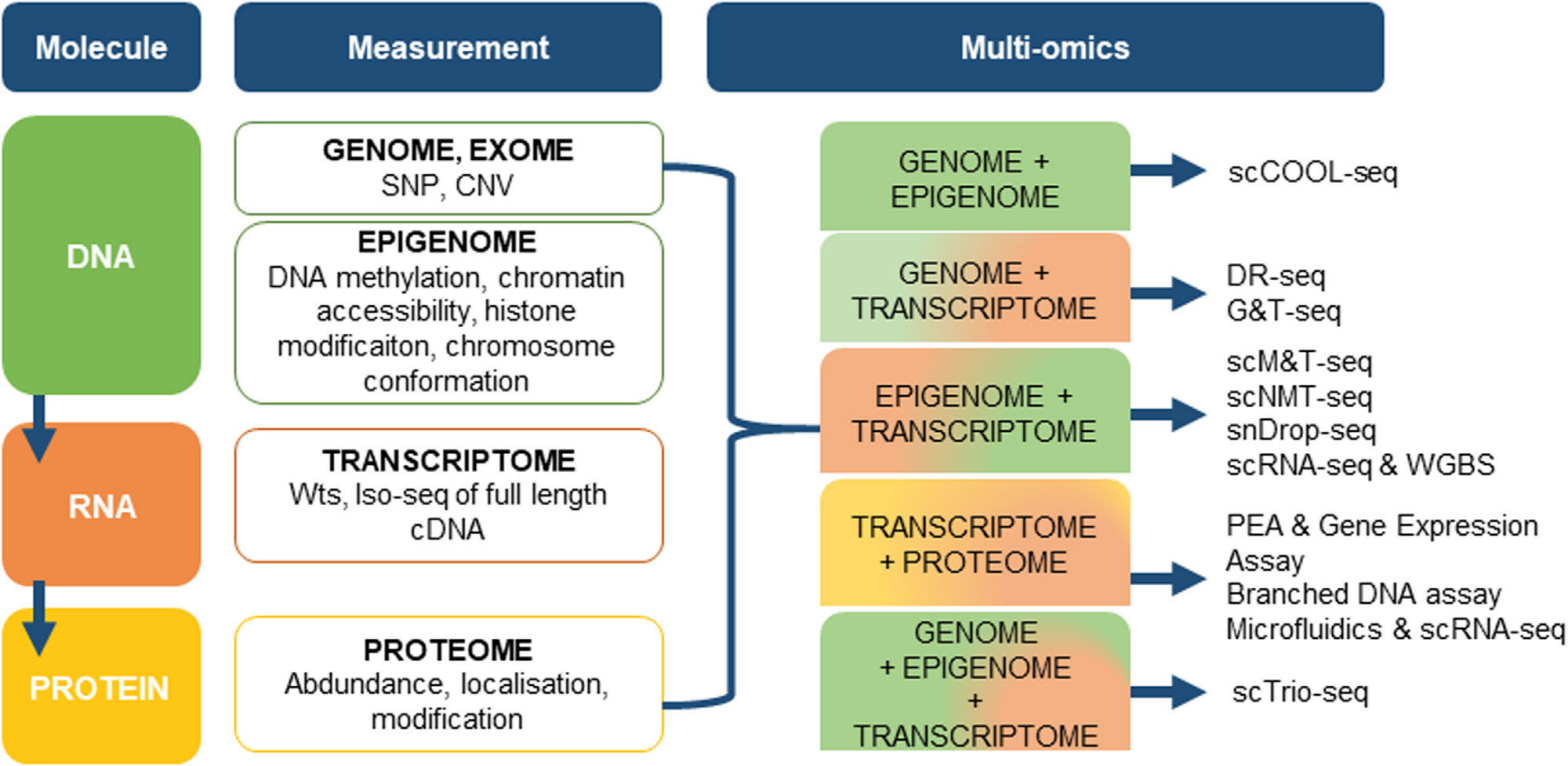
Multi‐omics of single‐cells: methods and applications. Based on the measurements that are of interest, a combination of single‐cell technologies are available. Left). The major types of molecules related to central biological dogma. Centre) Single‐cell measurements based on profiling the genome, epigenome, transcriptome, and proteome shown in different colors. Right) Single‐cell multi‐omics approaches based on the combination of different single‐cell sequencing methods to profile multiple molecule types of a single‐cell simultaneously.

Numerous methods have been developed to measure different “omes” at a single‐cell level, including DNA methylation, chromatin sequencing, and proteome analysis. Consequently, this enabled the growth of protocol by integrating existing cell sequencing methods (Figure 3). The standard workflow of single‐cell investigations begins with the isolation of single‐cells from a bulk sample. In this case the patient‐derived explant, followed by the isolation of multiple types of molecules from the same cell.^137^, ^138^. Next‐generation sequencing has allowed for genome‐wide analysis of DNA and RNA in single‐cells.^139^ Single‐cell RNA sequencing (scRNA‐seq) has emerged as a central tool for identifying and characterizing cell types and states, based on transcriptome profiling.^140^ The first single‐cell transcriptome analysis was reported in 2009, and many additional single‐cell RNA sequencing methods have been developed since, such as Quartz‐seq,^141^ smart‐seq (switching mechanism at 5′ end of the RNA transcript),^142^, ^143^ CEL‐seq (cell expression by linear amplification and sequencing)^144^ and more. However, successful scRNA‐seq of patient‐derived explants poses several challenges. First, obtaining fresh and viable tissue is extremely time‐sensitive and quick dissociation using enzymatic digestion can cause changes in gene expression or lead to loss of cells.^145^ Conversely, single‐nucleus RNA‐seq (snRNA‐seq) profiles single nuclei rather than single‐cells, enabling immediate sample processing for tissues that cannot be readily dissociated into a single‐cell suspension such as the brain, skeletal muscle, or adipose and frozen samples.^146^ This method also minimizes perturbations in gene expression associated with dissociation.^147^

Communication interactions are often too complex to predict reliably from the transcriptome alone as these networks are also directed by the proteins they code. Therefore, proteomic methods that measure protein abundance and state can provide quantification of ligands, receptors, downstream signaling molecules, and lineage‐specific transcription factors.^148^ This can be used to understand the origins of cellular heterogeneity.^149^, ^150^ Although quantifying proteins at single‐cell resolution is complicated by the transient function of proteins, single‐cell time‐of‐flight mass cytometry (CyTOF) is one method that can potentially address this.^151^ As posttranslational modifications (PTM) occur, CyTOF can be used to measure multiple signaling nodes via PTM‐specific antibody staining in a similar way to flow cytometry (FC) except metal isotopes are employed instead of fluorophores.^152^, ^153^, ^154^ Potentially this can be used to help identify crosstalk signaling between different cells by capturing ‘secrete‐able’ proteins. Additionally, strategies such as Disaggregation for Intracellular Signaling in Single Epithelial Cells from Tissue (DISSECT) have been developed to preserve native signaling for CyTOF applications.^155^ Experiments that include these approaches have revealed novel signaling relationships involved in cancer progression and drug resistance.^156^

Recent developments such as histoCAT, an open‐source computational toolbox, combines single‐cell mass cytometry, image analysis and novel algorithms for cell‐cell interaction network analysis. This can be used to define complex cell types and help elucidate patterns of cellular interactions within heterogeneous tissues.^157^ To interrogate solid tumor biology, it is necessary to scale and measure the expression of proteins. Recently, multiplexed proteome dynamics profiling (mPDP) was developed.^158^ This strategy combines quantitative liquid chromatography–tandem mass spectrometry (LC‐MS/MS) with dynamic stable isotope labelling by amino acids in cell culture (SILAC)^159^, ^160^ and enable the simultaneous analysis of changes in protein degradation and synthesis in a single experiment. LC‐MS/MS and SILAC can be used for secretome analysis and is of increasing interest as a potential source for biomarker discoveries and therapeutic targets.^161^ mPDP was previously employed to investigate interactions *in vitro* between epithelia and tumor and track secreted protein changes between the different cell types in the tumor microenvironment with their associated biological function.^162^

As the patient‐derived explant model provides the most patient‐relevant system to study cell–cell communication, secretome analysis gives insight on the interactions responsible for tumor growth and progression by defining the secreted proteins which were produced by the cells within the tumor microenvironment. However, the secretome is challenging to analyze due to the difficulties with sample collection and preparation. Various studies have tried to optimize their approaches to recover secreted proteins by using serum‐free media or attempted to determine the best protein precipitation and conjugation methods.^163^ Additionally, antibody arrays are often used as a complementary method to further validate secretory protein analysis.^164^

### 9.2 Combining the transcriptome and proteome

An ideal primary approach to construct cell‐cell communication networks is to focus on integrating the transcriptome and proteome. From an analytical perspective, the addition of multiple layers can reconstruct the entire outlook and provide insight to the intrinsic heterogeneity of single‐cells. Essentially, the central outlook is that cellular biology is highly heterogonous at all molecular levels. Most recently, two methods named CITE‐seq^165^ and REAP‐seq^166^ have been reported. Both methods follow a similar approach in which oligonucleotide‐labelled antibodies are used to integrate protein and transcriptome measurements producing a single‐cell readout. However, because the measurements of these two different molecules produced from the same gene may not directly correlate with one another, there is a need for multi‐modal analysis.^167^ This has led to the development of integrative computational frameworks such as CiteFuse.^168^ CiteFuse represents the first method to integrate both modalities of single‐cells in CITE‐seq data systematically. Using both simulations and an experimental CITE‐seq dataset generated from PBMCs.^169^ CiteFuse facilitated a more accurate identification of ligand‐receptor interactions compared to the expression of RNA alone (conventional approach). It was suggested that ligand‐receptor interactions identified through the conventional approach include false interactions due to high RNA expression that was not reciprocated in cell‐surface protein expression. Importantly, CiteFuse identified a fraction of interactions in each cluster that was not identified in the conventional approach. With the accumulating volume of multi‐omics data generated from CITE‐seq, CiteFuse is now currently freely available and implements a range of other tools for modality integration.^168^ It is important to note that CITE‐seq and REAP‐seq are currently limited to tagging cell‐surface markers and can only measure extracellular proteins and protein modifications, unlike CyTOF, which can additionally quantify intracellular targets.^170^ However, these two single‐cell sequencing techniques have provided a technique that increases measurable parameters compared to CyTOF as up to 50 metal isotopes are routinely used.^170^, ^171^ This provides deeper profiling to phosphoprotein networks which would be limited by employing CyTOF alone. Although sequencing‐based approaches suffer from technical variance, we emphasize that the combination of different single‐cell biotechnologies will optimize current understanding of cell‐cell communication.

### 9.3 Integrative methods for single‐cell multi‐omics technologies

#### 9.3.1 Computational resources: databases of interacting proteins

Recently developed CellPhoneDB, a public repository of ligands, receptors and their interactions enables a comprehensive, systematic analysis of cell‐cell communication molecules.^172^ This framework uses single‐cell transcriptomic data to consider the expression levels of ligands and receptors within each cell type and uses empirical shuffling to calculate which ligand‐receptor pairs display cell‐type specificity. Unlike most other databases, CellPhoneDB considers the subunit architecture of both ligands and receptors, reflecting heteromeric complexes accurately. An updated version of this resource recently incorporates additional functionalities to enable users to introduce new interacting molecules while reducing the time and resources needed to interrogate large data set.^173^ This provides a great advantage to annotate complex ligand‐receptor relationships using scRNA‐seq data. Future studies on CRC that incorporate scRNA‐seq with CyTOF and CellPhoneDB can potentially reveal novel relationships. Other optimization methods to determine cell‐cell relationships from single‐cell data analysis include SoptSC and online tools part of the FANTOM5 project.^174^, ^175^

#### 9.3.2 Modeling and assessing predicted interaction networks

Although single‐cell multi‐omics approaches would allow a holistic understanding of cellular functions by expression, function and identity, current research has not yet achieved this level of knowledge. Notably, most studies have focused on scRNA‐seq to characterize communication networks by ligand‐receptor interactions across all cell types in the tumor microenvironment. Recently, Chen and Mar applied single‐cell network modeling methods to single‐cell datasets in order to evaluate their capacity to identify known interaction networks these included, single‐cell regulatory network inference and clustering (SCENIC),^176^ SCODE,^177^ and partial information decomposition and context (PIDC).^178^, ^179^ Based on their comparisons, they reported that these network methods are not able to predict network structures from single‐cell expression data accurately. Effectively SCENIC is based on co‐expression network combined with bioinformatics knowledge, SCODE uses ordinary differential equations (ODEs), and PIDC is a mutual information‐based method. Essentially they found that single‐cell network inference methods did not have a high prediction accuracy and not surprisingly, the networks constructed by these methods were distinct from each other. This presents a challenge when predicting cell‐cell communication networks, based on the different models available, and results will vary. Although it is not the purpose of the review to go into details of these modelling methods, further investigation toward their predictability is necessary. Other bioinformatics tools are summarized in Table 4.

#### 9.3.3 Validating causal relationships

Finally, combining single‐cell multi‐omics data with perturbation experiments such as RNA interference (RNAi) ^180^, or CRISPR‐Cas9 ^148^, provides an efficient approach in verifying causal regulatory programs.^81^ Positive developments in high‐throughput gene technologies such as Perturb‐seq combine CRISPR/Cas9‐mediated gene perturbation with single‐cell sequencing ^181^ and have been reported to provide the same amount of causal information as RNAi or CRISPR/Cas9 mediated gene activation/deletions while also being less invasive. As single‐cell technologies continue to develop, the parameters that can be measured per cell will inevitably increase.^182^ Simultaneous measurements of multiple modalities from the same cell can help predict drug sensitivities in tumor cells, before any *in vivo* and/or *in vitro* drug testing.^183^

## 10 CONCLUSIONS

In summary, our view is that patient‐derived explant models will be much less variable compared to other *in vitro* and *in vivo* models when combined with CITE‐seq or scRNA‐seq, CyTOF, and CellPhoneDB towards study cell‐cell communication networks and compare differential responses to therapies in colorectal patients. These techniques can be used to compare different regions of the same patient‐derived explant as well as compare healthy to malignant tissue in identifying targeted receptors and their activated signaling pathway in response to specific ligands. This, together, can also significantly advance the clinical management of cancer as a powerful alternative for animal experiments to replacing and/or reducing animal use.

## AUTHOR CONTRIBUTIONS

A.S.N. designed the outline of the paper. S.A. and M.A. wrote the original draft preparation. S.A., M.A., and A.S.N. reviewed and edited the manuscript. A.S.N. supervised the study. All authors have read and approved the final version of this manuscript.

## CONFLICT OF INTEREST

The authors declare no potential conflicts of interest.

## Data Availability

Data sharing is not applicable to this article as no new data were created or analyzed in this study.

## References

[1] Rawla P , Sunkara T , Barsouk A . Epidemiology of colorectal cancer: incidence, mortality, survival, and risk factors. Prz Gastroenterol. 2019;14:89–103. 31616522 10.5114/pg.2018.81072 PMC6791134

[2] Wang W , Kandimalla R , Huang H , et al. Molecular subtyping of colorectal cancer: Recent progress, new challenges and emerging opportunities. Semin Cancer Biol. 2019;55:37–52. 29775690 10.1016/j.semcancer.2018.05.002 PMC6240404

[3] Grady WM , Carethers JM . Genomic and epigenetic instability in colorectal cancer pathogenesis. Gastroenterology. 2008;135:1079–1099. 18773902 10.1053/j.gastro.2008.07.076 PMC2866182

[4] Hermsen M , Postma C , Baak J , et al. Colorectal adenoma to carcinoma progression follows multiple pathways of chromosomal instability. Gastroenterology. 2002;123:1109–1119. 12360473 10.1053/gast.2002.36051

[5] Douglas EJ , Fiegler H , Rowan A , et al. Array comparative genomic hybridization analysis of colorectal cancer cell lines and primary carcinomas. Cancer Res. 2004;64:4817–4825. 15256451 10.1158/0008-5472.CAN-04-0328

[6] Martin ES , Tonon G , Sinha R , et al. Common and distinct genomic events in sporadic colorectal cancer and diverse cancer types. Cancer Res. 2007;67:10736–10743. 18006816 10.1158/0008-5472.CAN-07-2742

[7] Kuipers EJ , Grady WM , Lieberman D , et al. Colorectal cancer. Nature Reviews Disease Primers. 2015;1:15065. 10.1038/nrdp.2015.65 PMC4874655 27189416

[8] Sjoblom T , Jones S , Wood LD , et al. The consensus coding sequences of human breast and colorectal cancers. Science. 2006;314:268–274. 16959974 10.1126/science.1133427

[9] Fearon ER , Vogelstein B . A genetic model for colorectal tumorigenesis. Cell. 1990;61:759–767. 2188735 10.1016/0092-8674(90)90186-i

[10] Guinney J , Dienstmann R , Wang X , et al. The consensus molecular subtypes of colorectal cancer. Nat Med. 2015;21:1350–1356. 26457759 10.1038/nm.3967 PMC4636487

[11] Thanki K , Nicholls ME , Gujjar A , et al. Consensus molecular subtypes of colorectal cancer and their clinical implications. Int Biol Biomed J. 2017;3:105–111. 28825047 PMC5557054

[12] Sadanandam A , Gray J , Hanahan D . Reply to Colorectal cancer classification based on gene expression is not associated with FOLFIRI response. Nat Med. 2014;20:1231–1232. 25375919 10.1038/nm.3742

[13] Dienstmann R , Vermeulen L , Guinney J , Kopetz S , Tejpar S , Tabernero J . Consensus molecular subtypes and the evolution of precision medicine in colorectal cancer. Nature Reviews Cancer. 2017;17:79–92. 28050011 10.1038/nrc.2016.126

[14] Sveen A , Cremolini C , Dienstmann R . Predictive modeling in colorectal cancer: time to move beyond consensus molecular subtypes. Ann Oncol. 2019;30:1682–1685. 31868904 10.1093/annonc/mdz412

[15] Martinez‐Garcia R , Lopez‐Casas PP , Rico D , Valencia A , Hidalgo M . Colorectal cancer classification based on gene expression is not associated with FOLFIRI response. Nat Med. 2014;20:1230–1231. 25375918 10.1038/nm.3701

[16] Tape CJ . The heterocellular emergence of colorectal cancer. Trends Cancer. 2017;3:79–88. 28239669 10.1016/j.trecan.2016.12.004 PMC5312168

[17] Colangelo T , Polcaro G , Muccillo L , et al. Friend or foe? The tumour microenvironment dilemma in colorectal cancer. Biochim Biophys Acta Rev Cancer. 2017;1867:1–18. 27864070 10.1016/j.bbcan.2016.11.001

[18] Fiorini E , Veghini L , Corbo V . Modeling cell communication in cancer with organoids: making the complex simple. Front Cell Dev Biol. 2020;8:166. 32258040 10.3389/fcell.2020.00166 PMC7094029

[19] Karagiannis GS , Poutahidis T , Erdman SE , Kirsch R , Riddell RH , Diamandis EP . Cancer‐associated fibroblasts drive the progression of metastasis through both paracrine and mechanical pressure on cancer tissue. Mol Cancer Res. 2012;10:1403–1418. 23024188 10.1158/1541-7786.MCR-12-0307 PMC4399759

[20] Akieda Y , Ogamino S , Furuie H , et al. Cell competition corrects noisy Wnt morphogen gradients to achieve robust patterning in the zebrafish embryo. Nat Commun. 2019;10:4710. 31624259 10.1038/s41467-019-12609-4 PMC6797755

[21] Shamir ER , Ewald AJ . Three‐dimensional organotypic culture: experimental models of mammalian biology and disease. Nat Rev Mol Cell Biol. 2014;15:647–664. 25237826 10.1038/nrm3873 PMC4352326

[22] Yang H‐L , Thiyagarajan V , Shen P‐C , et al. Anti‐EMT properties of CoQ0 attributed to PI3K/AKT/NFKB/MMP‐9 signaling pathway through ROS‐mediated apoptosis. J Exp Clin Cancer Res. 2019;38:186. 31068208 10.1186/s13046-019-1196-x PMC6505074

[23] Farin HF , Jordens I , Mosa MH , et al. Visualization of a short‐range Wnt gradient in the intestinal stem‐cell niche. Nature. 2016;530:340–343. 26863187 10.1038/nature16937

[24] Lamouille S , Xu J , Derynck R . Molecular mechanisms of epithelial‐mesenchymal transition. Nat Rev Mol Cell Biol. 2014;15:178–196. 24556840 10.1038/nrm3758 PMC4240281

[25] Pérez‐Moreno MA , Locascio A , Rodrigo I , et al. A new role for E12/E47 in the repression of E‐cadherin expression and epithelial‐mesenchymal transitions. J Biol Chem. 2001;276:27424–27431. 11309385 10.1074/jbc.M100827200

[26] Wheelock MJ , Johnson KR . Cadherins as modulators of cellular phenotype. Annu Rev Cell Dev Biol. 2003;19:207–235. 14570569 10.1146/annurev.cellbio.19.011102.111135

[27] Wheelock MJ , Shintani Y , Maeda M , Fukumoto Y , Johnson KR . Cadherin switching. J Cell Sci. 2008;121:727–735. 18322269 10.1242/jcs.000455

[28] Islam S , Carey TE , Wolf GT , Wheelock MJ , Johnson KR . Expression of N‐cadherin by human squamous carcinoma cells induces a scattered fibroblastic phenotype with disrupted cell‐cell adhesion. J Cell Biol. 1996;135:1643–1654. 8978829 10.1083/jcb.135.6.1643 PMC2133960

[29] Guilford P . E‐cadherin downregulation in cancer: fuel on the fire?’ Mol Med Today. 1999;5:172–177. 10203750 10.1016/s1357-4310(99)01461-6

[30] Labernadie A , Kato T , Brugués A , et al. A mechanically active heterotypic E‐cadherin/N‐cadherin adhesion enables fibroblasts to drive cancer cell invasion. Nat Cell Biol. 2017;19:224–237. 28218910 10.1038/ncb3478 PMC5831988

[31] Li G , Satyamoorthy K , Herlyn M . N‐cadherin‐mediated intercellular interactions promote survival and migration of melanoma cells. Cancer Res. 2001;61:3819–3825. 11325858

[32] Hazan RB , Phillips GR , Qiao RF , Norton L , Aaronson SA . Exogenous expression of N‐cadherin in breast cancer cells induces cell migration, invasion, and metastasis. J Cell Biol. 2000;148:779–790. 10684258 10.1083/jcb.148.4.779 PMC2169367

[33] Fuhr L , Abrau M , Carbone A , et al. The Interplay between colon cancer cells and tumour‐associated stromal cells impacts the biological clock and enhances malignant phenotypes. Cancers (Basel). 2019;11:988. 10.3390/cancers11070988 PMC6678177 31311174

[34] Wei C , Yang C , Wang S , et al. Crosstalk between cancer cells and tumor associated macrophages is required for mesenchymal circulating tumor cell‐mediated colorectal cancer metastasis. Mol Cancer. 2019;18:64. 30927925 10.1186/s12943-019-0976-4 PMC6441214

[35] Pedrosa L , Esposito F , Thomson TM , Maurel J . The tumor microenvironment in colorectal cancer therapy. Cancers (Basel). 2019;11:1172. 10.3390/cancers11081172 PMC6721633 31416205

[36] Huang S , Chaudhary K , Garmire LX . More is better: recent progress in multi‐omics data integration methods. Front Genet. 2017;8:84. 28670325 10.3389/fgene.2017.00084 PMC5472696

[37] Vucic EA , Thu KL , Robison K , et al. Translating cancer ‘omics’ to improved outcomes. Genome Res. 2012;22:188–195. 22301133 10.1101/gr.124354.111 PMC3266027

[38] Yan H , Deng X , Chen H , et al. Identification of common and subtype‐specific mutated sub‐pathways for a cancer. Front Genet. 2019;10:1228. 31850075 10.3389/fgene.2019.01228 PMC6892778

[39] Avnet S , Lemma S , Cortini M , Di Pompo G , Perut F , Baldini N . Pre‐clinical models for studying the interaction between mesenchymal stromal cells and cancer cells and the induction of stemness. Front Oncol. 2019;9:305. 31114753 10.3389/fonc.2019.00305 PMC6502984

[40] Ammeux N , Housden BE , Georgiadis A , Hu Y , Perrimon N . Mapping signaling pathway cross‐talk in Drosophila cells. Proc Natl Acad Sci U S A. 2016;113:9940–9945. 27528688 10.1073/pnas.1610432113 PMC5024637

[41] Kakugawa S , Langton PF , Zebisch M , et al. Notum deacylates Wnt proteins to suppress signalling activity. Nature. 2015;519:187–192. 25731175 10.1038/nature14259 PMC4376489

[42] Inoue A , Deem AK , Kopetz S , Heffernan TP , Draetta GF , Carugo A . Current and future horizons of patient‐derived xenograft models in colorectal cancer translational research. Cancers (Basel). 2019;11:1321. 10.3390/cancers11091321 PMC6770280 31500168

[43] Qiu Y , Cai G , Zhou B , et al. A distinct metabolic signature of human colorectal cancer with prognostic potential. Clin Cancer Res. 2014;20:2136–2146. 24526730 10.1158/1078-0432.CCR-13-1939 PMC5902798

[44] Liu Y , Chen Y‐G . 2D‐ and 3D‐based intestinal stem cell cultures for personalized medicine. Cells. 2018;7:225. 10.3390/cells7120225 PMC6316377 30469504

[45] Pampaloni F , Reynaud EG , Stelzer EHK . The third dimension bridges the gap between cell culture and live tissue. Nat Rev Mol Cell Biol. 2007;8:839–845. 17684528 10.1038/nrm2236

[46] Baker BM , Chen CS . Deconstructing the third dimension: how 3D culture microenvironments alter cellular cues. J Cell Sci. 2012;125:3015–3024. 22797912 10.1242/jcs.079509 PMC3434846

[47] Bleijs M , Wetering M , Clevers H , Drost J . Xenograft and organoid model systems in cancer research. EMBO J. 2019;38:e101654–e101654. 31282586 10.15252/embj.2019101654 PMC6670015

[48] Hidalgo M , Bruckheimer E , Rajeshkumar NV , et al. A pilot clinical study of treatment guided by personalized tumorgrafts in patients with advanced cancer. Mol Cancer Ther. 2011;10:1311–1316. 21673092 10.1158/1535-7163.MCT-11-0233 PMC4629061

[49] Nakatsu G , Li X , Zhou H , et al. Gut mucosal microbiome across stages of colorectal carcinogenesis. Nat Commun. 2015;6:8727. 26515465 10.1038/ncomms9727 PMC4640069

[50] Feng Q , Liang S , Jia H , et al. Gut microbiome development along the colorectal adenoma‐carcinoma sequence. Nat Commun. 2015;6:6528. 25758642 10.1038/ncomms7528

[51] Cho S‐Y , et al. An integrative approach to precision cancer medicine using patient‐derived xenografts. Mol Cells. 2016;39:77–86. 26831452 10.14348/molcells.2016.2350 PMC4757806

[52] Julien S , Merino‐Trigo A , Lacroix L , et al. Characterization of a large panel of patient‐derived tumor xenografts representing the clinical heterogeneity of human colorectal cancer. Clin Cancer Res. 2012;18:5314–5328. 22825584 10.1158/1078-0432.CCR-12-0372

[53] Bardelli A , Corso S , Bertotti A , et al. Amplification of the MET receptor drives resistance to anti‐EGFR therapies in colorectal cancer. Cancer Discov. 2013;3:658–673. 23729478 10.1158/2159-8290.CD-12-0558 PMC4078408

[54] Lugli N , Dionellis VS , Ordóñez‐Morán P , et al. Enhanced rate of acquisition of point mutations in mouse intestinal adenomas compared to normal tissue. Cell Rep. 2017;19:2185–2192. 28614706 10.1016/j.celrep.2017.05.051

[55] Isella C , Terrasi A , Bellomo SE , et al. Stromal contribution to the colorectal cancer transcriptome. Nat Genet. 2015;47:312–319. 25706627 10.1038/ng.3224

[56] Voskoglou‐Nomikos T , Pater JL , Seymour L . Clinical predictive value of the in vitro cell line, human xenograft, and mouse allograft preclinical cancer models. Clin Cancer Res. 2003;9:4227–4239. 14519650

[57] Calon A , Lonardo E , Berenguer‐Llergo A , et al. Stromal gene expression defines poor‐prognosis subtypes in colorectal cancer. Nat Genet. 2015;47:320–329. 25706628 10.1038/ng.3225

[58] Rangarajan A , Weinberg RA . Opinion: comparative biology of mouse versus human cells: modelling human cancer in mice. Nat Rev Cancer. 2003;3:952–959. 14737125 10.1038/nrc1235

[59] Wang S , Gao D , Chen Y . The potential of organoids in urological cancer research. Nat Rev Urol. 2017;14:401–414. 28534535 10.1038/nrurol.2017.65 PMC5558053

[60] Sanz L , Cuesta ÁM , Salas C , Corbacho C , Bellas C , Álvarez‐Vallina L . Differential transplantability of human endothelial cells in colorectal cancer and renal cell carcinoma primary xenografts. Lab Invest. 2009;89:91–97. 19002108 10.1038/labinvest.2008.108

[61] Willey CD , Gilbert AN , Anderson JC , Gillespie GY . Patient‐derived xenografts as a model system for radiation research. Semin Radiat Oncol. 2015;25:273–280. 26384275 10.1016/j.semradonc.2015.05.008 PMC4758448

[62] Däster S , Amatruda N , Calabrese D , et al. Induction of hypoxia and necrosis in multicellular tumor spheroids is associated with resistance to chemotherapy treatment. Oncotarget. 2017;8:1725–1736. 27965457 10.18632/oncotarget.13857 PMC5352092

[63] Weiswald L‐B , Bellet D , Dangles‐Marie V . Spherical cancer models in tumor biology. Neoplasia. 2015;17:1–15. 25622895 10.1016/j.neo.2014.12.004 PMC4309685

[64] Ishiguro T , Ohata H , Sato Ai , Yamawaki K , Enomoto T , Okamoto K . Tumor‐derived spheroids: Relevance to cancer stem cells and clinical applications. Cancer Sci. 2017;108:283–289. 28064442 10.1111/cas.13155 PMC5378268

[65] Shaheen S , Ahmed M , Lorenzi F , Nateri AS . Spheroid‐formation (colonosphere) assay for in vitro assessment and expansion of stem cells in colon cancer. Stem Cell Rev. 2016;12:492–499. 10.1007/s12015-016-9664-6 PMC4919387 27207017

[66] Ricci‐Vitiani L , Lombardi DG , Pilozzi E , et al. Identification and expansion of human colon‐cancer‐initiating cells. Nature. 2007;445:111–115. 17122771 10.1038/nature05384

[67] O'brien CA , Pollett A , Gallinger S , Dick JE . A human colon cancer cell capable of initiating tumour growth in immunodeficient mice. Nature. 2007;445:106–110. 17122772 10.1038/nature05372

[68] Doctor A , Seifert V , Ullrich M , Hauser S , Pietzsch J . Three‐dimensional cell culture systems in radiopharmaceutical cancer research. Cancers. 2020;12:2765. 10.3390/cancers12102765 PMC7600608 32993034

[69] Edmondson R , Broglie JJ , Adcock AF , Yang L . Three‐dimensional cell culture systems and their applications in drug discovery and cell‐based biosensors. Assay Drug Dev Technol. 2014;12:207–218. 24831787 10.1089/adt.2014.573 PMC4026212

[70] Kawai S , Yamazaki M , Shibuya K , et al. Three‐dimensional culture models mimic colon cancer heterogeneity induced by different microenvironments. Sci Rep. 2020;10:3156. 32081957 10.1038/s41598-020-60145-9 PMC7035265

[71] Vermeulen L , Todaro M , De Sousa Mello F , et al. Single‐cell cloning of colon cancer stem cells reveals a multi‐lineage differentiation capacity. Proc Natl Acad Sci U S A. 2008;105:13427–13432. 18765800 10.1073/pnas.0805706105 PMC2533206

[72] Fang DD , Kim YJ , Lee CN , et al. Expansion of CD133+ colon cancer cultures retaining stem cell properties to enable cancer stem cell target discovery. Br J Cancer. 2010;102:1265–1275. 20332776 10.1038/sj.bjc.6605610 PMC2855999

[73] Stadler M , Scherzer M , Walter S , et al. Exclusion from spheroid formation identifies loss of essential cell‐cell adhesion molecules in colon cancer cells. Sci Rep. 2018;8:1151. 29348601 10.1038/s41598-018-19384-0 PMC5773514

[74] Kondo J , Endo H , Okuyama H , et al. Retaining cell‐cell contact enables preparation and culture of spheroids composed of pure primary cancer cells from colorectal cancer. Proc Natl Acad Sci U S A. 2011;108:6235–6240. 21444794 10.1073/pnas.1015938108 PMC3076886

[75] Torras N , García‐Díaz M , Fernández‐Majada V , Martínez E . Mimicking epithelial tissues in three‐dimensional cell culture models. Front Bioeng Biotechnol. 2018;6:197. 30619844 10.3389/fbioe.2018.00197 PMC6305315

[76] Cui X , Hartanto Y , Zhang H . Advances in multicellular spheroids formation. J R Soc Interface. 20160877, 2017;14. 28202590 10.1098/rsif.2016.0877 PMC5332573

[77] Sato T , Vries RG , Snippert HJ , et al. Single Lgr5 stem cells build crypt‐villus structures in vitro without a mesenchymal niche. Nature. 2009;459:262–265. 19329995 10.1038/nature07935

[78] Li N , Babaei‐Jadidi R , Lorenzi F , et al. An FBXW7‐ZEB2 axis links EMT and tumour microenvironment to promote colorectal cancer stem cells and chemoresistance. Oncogenesis. 2019;8:13. 30783098 10.1038/s41389-019-0125-3 PMC6381143

[79] Xu H , Jiao Y , Qin S , Zhao W , Chu Q , Wu K . Organoid technology in disease modelling, drug development, personalized treatment and regeneration medicine. Exp Hematol Oncol. 2018;7:30. 30534474 10.1186/s40164-018-0122-9 PMC6282260

[80] Li X , Nadauld L , Ootani A , et al. Oncogenic transformation of diverse gastrointestinal tissues in primary organoid culture. Nat Med. 2014;20:769–777. 24859528 10.1038/nm.3585 PMC4087144

[81] Kashfi H , Jinks N , Nateri AS . Generating and utilizing murine Cas9‐expressing intestinal organoids for large‐scale knockout genetic screening. Methods Mol Biol. 2020;2171:257–269. 32705648 10.1007/978-1-0716-0747-3_17

[82] Fiorini E , Veghini L , Corbo V . Modeling cell communication in cancer with organoids: making the complex simple. Front Cell Dev Biol. 2020;8:166–166. 32258040 10.3389/fcell.2020.00166 PMC7094029

[83] Szvicsek Z , Oszvald Á , Szabó L , et al. Extracellular vesicle release from intestinal organoids is modulated by Apc mutation and other colorectal cancer progression factors. Cell Mol Life Sci. 2019;76:2463–2476. 31028424 10.1007/s00018-019-03052-1 PMC6529386

[84] Rahmani S , Breyner NM , Su H‐M , Verdu EF , Didar TF . Intestinal organoids: a new paradigm for engineering intestinal epithelium in vitro. Biomaterials. 2019;194:195–214. 30612006 10.1016/j.biomaterials.2018.12.006

[85] Kim J , Koo B‐K , Knoblich JA . Human organoids: model systems for human biology and medicine. Nat Rev Mol Cell Biol. 2020;21:571–584. 32636524 10.1038/s41580-020-0259-3 PMC7339799

[86] Sato T , Stange DE , Ferrante M , et al. Long‐term Expansion of epithelial organoids from human colon, adenoma, adenocarcinoma, and Barrett's epithelium. Gastroenterology. 2011;141:1762–1772. 21889923 10.1053/j.gastro.2011.07.050

[87] Kimlin LC , Casagrande G , Virador VM . In vitro three‐dimensional (3D) models in cancer research: an update. Mol Carcinog. 2013;52:167–182. 22162252 10.1002/mc.21844

[88] Majumder B , Baraneedharan U , Thiyagarajan S , et al. Predicting clinical response to anticancer drugs using an ex vivo platform that captures tumour heterogeneity. Nat Commun. 2015;6:6169. 25721094 10.1038/ncomms7169 PMC4351621

[89] Yamada KM , Cukierman E . Modeling tissue morphogenesis and cancer in 3D. Cell. 2007;130:601–610. 17719539 10.1016/j.cell.2007.08.006

[90] Wilson SS , Tocchi A , Holly MK , Parks WC , Smith JG . A small intestinal organoid model of non‐invasive enteric pathogen‐epithelial cell interactions. Mucosal Immunol. 2015;8:352–361. 25118165 10.1038/mi.2014.72 PMC4326599

[91] Mithal A , Capilla A , Heinze D , et al. Generation of mesenchyme free intestinal organoids from human induced pluripotent stem cells. Nat Commun. 2020;11:215. 31924806 10.1038/s41467-019-13916-6 PMC6954238

[92] Langhans SA . Three‐dimensional in vitro cell culture models in drug discovery and drug repositioning. Front Pharmacol. 2018;9:6. 29410625 10.3389/fphar.2018.00006 PMC5787088

[93] Marusyk A , Tabassum DP , Janiszewska M , et al. Spatial proximity to fibroblasts impacts molecular features and therapeutic sensitivity of breast cancer cells influencing clinical outcomes. Cancer Res. 2016;76:6495–6506. 27671678 10.1158/0008-5472.CAN-16-1457 PMC5344673

[94] Lee GY , Kenny PA , Lee EH , Bissell MJ . Three‐dimensional culture models of normal and malignant breast epithelial cells. Nat Methods. 2007;4:359–365. 17396127 10.1038/nmeth1015 PMC2933182

[95] Dijkstra KK , Cattaneo CM , Weeber F , et al. Generation of tumor‐reactive T cells by co‐culture of peripheral blood lymphocytes and tumor organoids. Cell. 2018;174:1586–1598.e12. 30100188 10.1016/j.cell.2018.07.009 PMC6558289

[96] Neal JT , Li X , Zhu J , et al. Organoid modeling of the tumor immune microenvironment. Cell. 2018;175:1972–1988.e16. 30550791 10.1016/j.cell.2018.11.021 PMC6656687

[97] Bar‐Ephraim YE , Kretzschmar K , Clevers H . Organoids in immunological research. Nat Rev Immunol. 2020;20:279–293. 31853049 10.1038/s41577-019-0248-y

[98] Kong JCH , Guerra GR , Millen RM , et al. Tumor‐infiltrating lymphocyte function predicts response to neoadjuvant chemoradiotherapy in locally advanced rectal cancer. JCO Precision Oncology. 2018;2:1–15. 35135158 10.1200/PO.18.00075

[99] Qin X , Sufi J , Vlckova P , et al. Cell‐type‐specific signaling networks in heterocellular organoids. Nat Methods. 2020;17:335–342. 32066960 10.1038/s41592-020-0737-8 PMC7060080

[100] Devarasetty M , Dominijanni A , Herberg S , Shelkey E , Skardal A , Soker S . Simulating the human colorectal cancer microenvironment in 3D tumor‐stroma co‐cultures in vitro and in vivo. Sci Rep. 2020;10:9832. 32555362 10.1038/s41598-020-66785-1 PMC7300090

[101] Bauleth‐Ramos T , Feijão T , Gonçalves A , et al. Colorectal cancer triple co‐culture spheroid model to assess the biocompatibility and anticancer properties of polymeric nanoparticles. J Control Release. 2020;323:398–411. 32320816 10.1016/j.jconrel.2020.04.025

[102] Courau T , Bonnereau J , Chicoteau J , et al. Cocultures of human colorectal tumor spheroids with immune cells reveal the therapeutic potential of MICA/B and NKG2A targeting for cancer treatment. J Immunother Cancer. 2019;7:74. 30871626 10.1186/s40425-019-0553-9 PMC6417026

[103] Huang R , Zhang D , Li F , et al. Loss of Fas expression and high expression of HLA‐E promoting the immune escape of early colorectal cancer cells. Oncol Lett. 2017;13:3379–3386. 28521443 10.3892/ol.2017.5891 PMC5431327

[104] Hoffmann OI , Ilmberger C , Magosch S , Joka M , Jauch K‐W , Mayer B . Impact of the spheroid model complexity on drug response. J Biotechnol. 2015;205:14–23. 25746901 10.1016/j.jbiotec.2015.02.029

[105] Tuveson D , Clevers H . Cancer modeling meets human organoid technology. Science. 2019;364:952–955. 31171691 10.1126/science.aaw6985

[106] Hanahan D , Weinberg RA . Hallmarks of cancer: the next generation. Cell. 2011;144:646–674. 21376230 10.1016/j.cell.2011.02.013

[107] Kobayashi H , Enomoto A , Woods SL , Burt AD , Takahashi M , Worthley DL . Cancer‐associated fibroblasts in gastrointestinal cancer. Nat Rev Gastroenterol Hepatol. 2019;16:282–295. 30778141 10.1038/s41575-019-0115-0

[108] Cattaneo CM , Dijkstra KK , Fanchi LF , et al. Tumor organoid–T‐cell coculture systems. Nat Protoc. 2020;15:15–39. 31853056 10.1038/s41596-019-0232-9 PMC7610702

[109] Karekla E , Liao W‐J , Sharp B , et al. *Ex Vivo* explant cultures of non–small cell lung carcinoma enable evaluation of primary tumor responses to anticancer therapy. Cancer Res.. 2017;77:2029–2039. 28202521 10.1158/0008-5472.CAN-16-1121

[110] Ahmed M , Jinks N , Babaei‐Jadidi R , et al. Repurposing antibacterial AM404 as a potential anticancer drug for targeting colorectal cancer stem‐like cells. Cancers. 2019;12:106. 10.3390/cancers12010106 PMC7017077 31906201

[111] Centenera MM , Hickey TE , Jindal S , et al. A patient‐derived explant (PDE) model of hormone‐dependent cancer. Mol Oncol. 2018;12:1608–1622. 30117261 10.1002/1878-0261.12354 PMC6120230

[112] Sönnichsen R , Hennig L , Blaschke V , et al. Individual susceptibility analysis using patient‐derived slice cultures of colorectal carcinoma. Clin Colorectal Cancer. 2018;17:e189–e199. 29233603 10.1016/j.clcc.2017.11.002

[113] Centenera MM , Raj GV , Knudsen KE , Tilley WD , Butler LM . Ex vivo culture of human prostate tissue and drug development. Nat Rev Urol. 2013;10:483–487. 23752995 10.1038/nrurol.2013.126

[114] Powley IR , Patel M , Miles G , et al. Patient‐derived explants (PDEs) as a powerful preclinical platform for anti‐cancer drug and biomarker discovery. Br J Cancer. 2020;122:735–744. 31894140 10.1038/s41416-019-0672-6 PMC7078311

[115] Freeman AE , Hoffman RM . In vivo‐like growth of human tumors in vitro. Proc Natl Acad Sci U S A. 1986;83:2694–2698. 3458228 10.1073/pnas.83.8.2694 PMC323366

[116] Shafi AA , Schiewer MJ , De Leeuw R , et al. Patient‐derived models reveal impact of the tumor microenvironment on therapeutic response. Eur Urol Oncol. 2018;1:325–337. 30467556 10.1016/j.euo.2018.04.019 PMC6241309

[117] Collins A , Miles GJ , Wood J , Macfarlane M , Pritchard C , Moss E . Patient‐derived explants, xenografts and organoids: 3‐dimensional patient‐relevant pre‐clinical models in endometrial cancer. Gynecol Oncol. 2020;156:251–259. 31767187 10.1016/j.ygyno.2019.11.020

[118] O'toole A , Michielsen AJ , Nolan B , et al. Tumour microenvironment of both early‐ and late‐stage colorectal cancer is equally immunosuppressive. Br J Cancer. 2014;111:927–932. 25058349 10.1038/bjc.2014.367 PMC4150274

[119] Michielsen AJ , Hogan AE , Marry J , et al. Tumour tissue microenvironment can inhibit dendritic cell maturation in colorectal cancer. PLoS One. 2011;6:e27944. 22125641 10.1371/journal.pone.0027944 PMC3220715

[120] Halama N , Zoernig I , Berthel A , et al. Tumoral immune cell exploitation in colorectal cancer metastases can be targeted effectively by anti‐CCR5 therapy in cancer patients. Cancer Cell. 2016;29:587–601. 27070705 10.1016/j.ccell.2016.03.005

[121] Risbridger GP , Toivanen R , Taylor RA . Preclinical models of prostate cancer: patient‐derived xenografts, organoids, and other explant models. Cold Spring Harb Perspect Med. 2018;8:a030536. 29311126 10.1101/cshperspect.a030536 PMC6071547

[122] Pauli C , Hopkins BD , Prandi D , et al. Personalized in vitro and in vivo cancer models to guide precision medicine. Cancer Discov. 2017;7:462–477. 28331002 10.1158/2159-8290.CD-16-1154 PMC5413423

[123] Blencowe M , Arneson D , Ding J , Chen YW , Saleem Z , Yang X . Network modeling of single‐cell omics data: challenges, opportunities, and progresses. Emerg Top Life Sci. 2019;3:379–398. 32270049 10.1042/ETLS20180176 PMC7141415

[124] Langfelder P , Horvath S . WGCNA: an R package for weighted correlation network analysis. BMC Bioinformatics. 2008;9:559. 19114008 10.1186/1471-2105-9-559 PMC2631488

[125] Zhang B , Horvath S . A general framework for weighted gene co‐expression network analysis. Stat Appl Genet Mol Biol. 2005;4:Article17. 16646834 10.2202/1544-6115.1128

[126] Ayiomamitis GD , Notas G , Vasilakaki T , et al. Understanding the interplay between COX‐2 and hTERT in colorectal cancer using a multi‐omics analysis. Cancers (Basel). 2019;11:1536. 10.3390/cancers11101536 PMC6827032 31614548

[127] Wendum D , Comperat E , Boëlle P‐Y , et al. Cytoplasmic phospholipase A2 alpha overexpression in stromal cells is correlated with angiogenesis in human colorectal cancer. Mod Pathol. 2005;18:212–220. 15475936 10.1038/modpathol.3800284

[128] Jahangiri B , Khalaj‐Kondori M , Asadollahi E , Sadeghizadeh M . Cancer‐associated fibroblasts enhance cell proliferation and metastasis of colorectal cancer SW480 cells by provoking long noncoding RNA UCA1. J Cell Commun Signal. 2019;13:53–64. 29948578 10.1007/s12079-018-0471-5 PMC6381368

[129] Nielsen DL , Palshof JA , Larsen FO , Jensen BV , Pfeiffer P . A systematic review of salvage therapy to patients with metastatic colorectal cancer previously treated with fluorouracil, oxaliplatin and irinotecan +/‐ targeted therapy. Cancer Treat Rev. 2014;40:701–715. 24731471 10.1016/j.ctrv.2014.02.006

[130] Guo AJ , Wang FJ , Ji Q , et al. Proteome analyses reveal S100A11, S100P, and RBM25 are tumor biomarkers in colorectal cancer. Proteomics Clin Appl. 2020;15:2000056. 10.1002/prca.202000056 33098374

[131] Sever R , Brugge JS . Signal transduction in cancer. Cold Spring Harb Perspect Med. 2015;5:a006098. 25833940 10.1101/cshperspect.a006098 PMC4382731

[132] Brulé SY , Jonker DJ , Karapetis CS , et al. Location of colon cancer (right‐sided versus left‐sided) as a prognostic factor and a predictor of benefit from cetuximab in NCIC CO.17. Eur J Cancer. 2015;51:1405–1414. 25979833 10.1016/j.ejca.2015.03.015

[133] Hu W , Yang Y , Li X , et al. Multi‐omics approach reveals distinct differences in left‐ and right‐sided colon cancer. Mol Cancer Res. 2018;16:476–485. 29187560 10.1158/1541-7786.MCR-17-0483

[134] Koelzer VH , Lugli A , Dawson H , et al. CD8/CD45RO T‐cell infiltration in endoscopic biopsies of colorectal cancer predicts nodal metastasis and survival. J Transl Med. 2014;12:81. 24679169 10.1186/1479-5876-12-81 PMC4022053

[135] Park JH , Wyk H , Mcmillan DC , et al. Preoperative, biopsy‐based assessment of the tumour microenvironment in patients with primary operable colorectal cancer. J Pathol Clin Res. 2020;6:30–39. 31486287 10.1002/cjp2.143 PMC6966701

[136] Kemp Bohan PM , Chick RC , Hickerson AT , et al. Correlation of tumor microenvironment from biopsy and resection specimens in untreated colorectal cancer patients: a surprising lack of agreement. Cancer Immunol Immunother. 2020. 10.1007/s00262-020-02784-5 PMC7658304 33180182

[137] Macaulay IC , Haerty W , Kumar P , et al. G&T‐seq: parallel sequencing of single‐cell genomes and transcriptomes. Nat Methods. 2015;12:519–522. 25915121 10.1038/nmeth.3370

[138] Angermueller C , Clark SJ , Lee HJ , et al. Parallel single‐cell sequencing links transcriptional and epigenetic heterogeneity. Nat Methods. 2016;13:229–232. 26752769 10.1038/nmeth.3728 PMC4770512

[139] Tang F , Barbacioru C , Wang Y , et al. mRNA‐Seq whole‐transcriptome analysis of a single cell. Nat Methods. 2009;6:377–382. 19349980 10.1038/nmeth.1315

[140] Klein AM , Mazutis L , Akartuna I , et al. Droplet barcoding for single‐cell transcriptomics applied to embryonic stem cells. Cell. 2015;161:1187–1201. 26000487 10.1016/j.cell.2015.04.044 PMC4441768

[141] Sasagawa Y , Nikaido I , Hayashi T , et al. Quartz‐Seq: a highly reproducible and sensitive single‐cell RNA sequencing method, reveals non‐genetic gene‐expression heterogeneity. Genome Biol. 2013;14:R31. 23594475 10.1186/gb-2013-14-4-r31 PMC4054835

[142] Goetz JJ , Trimarchi JM . Transcriptome sequencing of single cells with Smart‐Seq. Nat Biotechnol. 2012;30:763–765. 22871714 10.1038/nbt.2325

[143] Picelli S , Faridani OR , Björklund ÅK , Winberg G , Sagasser S , Sandberg R . Full‐length RNA‐seq from single cells using Smart‐seq2. Nat Protoc. 2014;9:171–181. 24385147 10.1038/nprot.2014.006

[144] Hashimshony T , Wagner F , Sher N , Yanai I . CEL‐Seq: single‐cell RNA‐Seq by multiplexed linear amplification. Cell Rep. 2012;2:666–673. 22939981 10.1016/j.celrep.2012.08.003

[145] Slyper M , Porter CBM , Ashenberg O , et al. A single‐cell and single‐nucleus RNA‐Seq toolbox for fresh and frozen human tumors. Nat Med. 2020;26:792–802. 32405060 10.1038/s41591-020-0844-1 PMC7220853

[146] Ding J , Adiconis X , Simmons SK , et al. Systematic comparison of single‐cell and single‐nucleus RNA‐sequencing methods. Nat Biotechnol. 2020;38:737–746. 32341560 10.1038/s41587-020-0465-8 PMC7289686

[147] Van Den Brink SC , Sage F , Vértesy Á , et al. Single‐cell sequencing reveals dissociation‐induced gene expression in tissue subpopulations. Nat Methods. 2017;14:935–936. 28960196 10.1038/nmeth.4437

[148] Efremova M , Teichmann SA . Computational methods for single‐cell omics across modalities. Nat Methods. 2020;17:14–17. 31907463 10.1038/s41592-019-0692-4

[149] Dalerba P , Kalisky T , Sahoo D , et al. Single‐cell dissection of transcriptional heterogeneity in human colon tumors. Nat Biotechnol. 2011;29:1120–1127. 22081019 10.1038/nbt.2038 PMC3237928

[150] Ma C , Cheung AF , Chodon T , et al. Multifunctional T‐cell analyses to study response and progression in adoptive cell transfer immunotherapy. Cancer Discov. 2013;3:418–429. 23519018 10.1158/2159-8290.CD-12-0383 PMC3716460

[151] Bendall SC , Simonds EF , Qiu P , et al. Single‐cell mass cytometry of differential immune and drug responses across a human hematopoietic continuum. Science. 2011;332:687–696. 21551058 10.1126/science.1198704 PMC3273988

[152] Irish JM , Hovland R , Krutzik PO , et al. Single cell profiling of potentiated phospho‐protein networks in cancer cells. Cell. 2004;118:217–228. 15260991 10.1016/j.cell.2004.06.028

[153] Tape CJ . Systems biology analysis of heterocellular signaling. Trends Biotechnol. 2016;34:627–637. 27087613 10.1016/j.tibtech.2016.02.016

[154] Di Palma S , Bodenmiller B . Unraveling cell populations in tumors by single‐cell mass cytometry. Curr Opin Biotechnol. 2015;31:122–129. 25123841 10.1016/j.copbio.2014.07.004

[155] Simmons AJ , Banerjee A , Mckinley ET , et al. Cytometry‐based single‐cell analysis of intact epithelial signaling reveals MAPK activation divergent from TNF‐α‐induced apoptosis in vivo. Mol Syst Biol. 2015;11:835. 26519361 10.15252/msb.20156282 PMC4631206

[156] Lun X‐K , Szklarczyk D , Gábor A , et al. Analysis of the human kinome and phosphatome by mass cytometry reveals overexpression‐induced effects on cancer‐related signaling. Mol Cell. 2019;74:1086–1102.e5. 31101498 10.1016/j.molcel.2019.04.021 PMC6561723

[157] Schapiro D , Jackson HW , Raghuraman S , et al. histoCAT: analysis of cell phenotypes and interactions in multiplex image cytometry data. Nat Methods. 2017;14:873–876. 28783155 10.1038/nmeth.4391 PMC5617107

[158] Savitski MM , Zinn N , Faelth‐Savitski M , et al. Multiplexed proteome dynamics profiling reveals mechanisms controlling protein homeostasis. Cell. 2018;173:260–274.e25. 29551266 10.1016/j.cell.2018.02.030 PMC5871718

[159] Doherty MK , Hammond DE , Clague MJ , Gaskell SJ , Beynon RJ . Turnover of the human proteome: determination of protein intracellular stability by dynamic SILAC. J Proteome Res. 2009;8:104–112. 18954100 10.1021/pr800641v

[160] Schwanhäusser B , Busse D , Li N , et al. Corrigendum: Global quantification of mammalian gene expression control. Nature. 2013;495:126–127. 23407496 10.1038/nature11848

[161] Paré B , Deschênes LT , Pouliot R , Dupré N , Gros‐Louis F . An optimized approach to recover secreted proteins from fibroblast conditioned‐media for secretomic analysis. Front Cell Neurosci. 2016;10:70. 27064649 10.3389/fncel.2016.00070 PMC4814560

[162] Zeng X , Yang P , Chen B , et al. Quantitative secretome analysis reveals the interactions between epithelia and tumor cells by in vitro modulating colon cancer microenvironment. J Proteomics. 2013;89:51–70. 23748022 10.1016/j.jprot.2013.05.032

[163] Brandi J , Manfredi M , Speziali G , Gosetti F , Marengo E , Cecconi D . Proteomic approaches to decipher cancer cell secretome. Semin Cell Dev Biol. 2018;78:93–101. 28684183 10.1016/j.semcdb.2017.06.030

[164] Sharma A , Bender S , Zimmermann M , Riesterer O , Broggini‐Tenzer A , Pruschy MN . Secretome signature identifies ADAM17 as novel target for radiosensitization of non‐small cell lung cancer. Clin Cancer Res. 2016;22:4428–4439. 27076628 10.1158/1078-0432.CCR-15-2449

[165] Stoeckius M , Hafemeister C , Stephenson W , et al. Simultaneous epitope and transcriptome measurement in single cells. Nat Methods. 2017;14:865–868. 28759029 10.1038/nmeth.4380 PMC5669064

[166] Peterson VM , Zhang KXi , Kumar N , et al. Multiplexed quantification of proteins and transcripts in single cells. Nat Biotechnol. 2017;35:936–939. 28854175 10.1038/nbt.3973

[167] See P , Lum J , Chen J , Ginhoux F . A single‐cell sequencing guide for immunologists. Front Immunol. 2018;9:2425. 30405621 10.3389/fimmu.2018.02425 PMC6205970

[168] Kim HJ , Lin Y , Geddes TA , Yang JYH , Yang P . CiteFuse enables multi‐modal analysis of CITE‐seq data. Bioinformatics. 2020;36:4137–4143. 32353146 10.1093/bioinformatics/btaa282

[169] Mimitou EP , Cheng A , Montalbano A , et al. Multiplexed detection of proteins, transcriptomes, clonotypes and CRISPR perturbations in single cells. Nat Methods. 2019;16:409–412. 31011186 10.1038/s41592-019-0392-0 PMC6557128

[170] Lun X‐K , Bodenmiller B . Profiling cell signaling networks at single‐cell resolution. Mol Cell Proteomics. 2020;19:744–756. 10.1074/mcp.R119.001790 PMC7196580 32132232

[171] Hartmann FJ , Simonds EF , Vivanco N , et al. Scalable conjugation and characterization of immunoglobulins with stable mass isotope reporters for single‐cell mass cytometry analysis. Methods Mol Biol. 2019;1989:55–81. 31077099 10.1007/978-1-4939-9454-0_5 PMC6687300

[172] Vento‐Tormo R , Efremova M , Botting RA , et al. Single‐cell reconstruction of the early maternal‐fetal interface in humans. Nature. 2018;563:347–353. 30429548 10.1038/s41586-018-0698-6 PMC7612850

[173] Efremova M , Vento‐Tormo M , Teichmann SA , Vento‐Tormo R . CellPhoneDB: inferring cell‐cell communication from combined expression of multi‐subunit ligand‐receptor complexes. Nat Protoc. 2020;15:1484–1506. 32103204 10.1038/s41596-020-0292-x

[174] Wang S , Karikomi M , Maclean AL , Nie Q . Cell lineage and communication network inference via optimization for single‐cell transcriptomics. Nucleic Acids Res. 2019;47:e66. 30923815 10.1093/nar/gkz204 PMC6582411

[175] Ramilowski JA , Goldberg T , Harshbarger J , et al. A draft network of ligand‐receptor‐mediated multicellular signalling in human. Nat Commun. 2015;6:7866. 26198319 10.1038/ncomms8866 PMC4525178

[176] Aibar S , González‐Blas CB , Moerman T , et al. SCENIC: single‐cell regulatory network inference and clustering. Nat Methods. 2017;14:1083–1086. 28991892 10.1038/nmeth.4463 PMC5937676

[177] Matsumoto H , Kiryu H , Furusawa C , et al. SCODE: an efficient regulatory network inference algorithm from single‐cell RNA‐Seq during differentiation. Bioinformatics. 2017;33:2314–2321. 28379368 10.1093/bioinformatics/btx194 PMC5860123

[178] Chen S , Mar JC . Evaluating methods of inferring gene regulatory networks highlights their lack of performance for single cell gene expression data. BMC Bioinformatics. 2018;19:232. 29914350 10.1186/s12859-018-2217-z PMC6006753

[179] Filippi S , Holmes CC , Nieto‐Barajas LE . Scalable Bayesian nonparametric measures for exploring pairwise dependence via Dirichlet Process Mixtures. Electron J Stat. 2016;10:3338–3354. 29707100 10.1214/16-ejs1171 PMC5915294

[180] Duan L , Zhang X‐Di , Miao W‐Y , et al. PDGFRβ cells rapidly relay inflammatory signal from the circulatory system to neurons via chemokine CCL2. Neuron. 2018;100:183–200.e8. 30269986 10.1016/j.neuron.2018.08.030

[181] Dixit A , Parnas O , Li B , et al. Perturb‐seq: dissecting molecular circuits with scalable single‐cell RNA profiling of pooled genetic screens. Cell. 2016;167:1853–1866.e17. 27984732 10.1016/j.cell.2016.11.038 PMC5181115

[182] Stuart T , Satija R . Integrative single‐cell analysis. Nat Rev Genet. 2019;20:257–272. 30696980 10.1038/s41576-019-0093-7

[183] Ma A , Mcdermaid A , Xu J , Chang Y , Ma Q . Integrative methods and practical challenges for single‐cell multi‐omics. Trends Biotechnol. 2020;38:1007–1022,. 32818441 10.1016/j.tibtech.2020.02.013 PMC7442857

[184] Kapałczyńska M , Kolenda T , Przybyła W , et al. 2D and 3D cell cultures ‐ a comparison of different types of cancer cell cultures. Arch Med Sci. 2018;14:910–919. 30002710 10.5114/aoms.2016.63743 PMC6040128

[185] Grässer U , Bubel M , Sossong D , Oberringer M , Pohlemann T , Metzger W . Dissociation of mono‐ and co‐culture spheroids into single cells for subsequent flow cytometric analysis. Ann Anat. 2018;216:1–8. 29162481 10.1016/j.aanat.2017.10.002

[186] Noben M , Vanhove W , Arnauts K , et al. Human intestinal epithelium in a dish: Current models for research into gastrointestinal pathophysiology. United European Gastroenterol J. 2017;5:1073–1081. 10.1177/2050640617722903 PMC5721984 29238585

[187] Xu H , Jiao Y , Qin S , Zhao W , Chu Q , Wu K . Organoid technology in disease modelling, drug development, personalized treatment and regeneration medicine. Experimental Hematology, Oncology. 2018;7:30–30. 30534474 10.1186/s40164-018-0122-9 PMC6282260

[188] Van De Wetering M , Francies HE , Francis JM , et al. Prospective derivation of a living organoid biobank of colorectal cancer patients. Cell. 2015;161:933–945. 25957691 10.1016/j.cell.2015.03.053 PMC6428276

[189] Tignanelli CJ , Loeza SGH , Yeh JJ . KRAS and PIK3CA mutation frequencies in patient‐derived xenograft models of pancreatic and colorectal cancer are reflective of patient tumors and stable across passages. Am Surg. 2014;80:873–877. 25197873 PMC4425299

[190] Miki Y , Ono K , Hata S , Suzuki T , Kumamoto H , Sasano H . The advantages of co‐culture over mono cell culture in simulating in vivo environment. J Steroid Biochem Mol Biol. 2012;131:68–75. 22265957 10.1016/j.jsbmb.2011.12.004

[191] Cirri P , Chiarugi P . Cancer associated fibroblasts: the dark side of the coin. Am J Cancer Res. 2011;1:482–497. 21984967 PMC3186047

[192] Hawinkels LJAC , Paauwe M , Verspaget HW , et al. Interaction with colon cancer cells hyperactivates TGF‐β signaling in cancer‐associated fibroblasts. Oncogene. 2014;33:97–107. 23208491 10.1038/onc.2012.536

[193] Malfettone A , Silvestris N , Saponaro C , et al. High density of tryptase‐positive mast cells in human colorectal cancer: a poor prognostic factor related to protease‐activated receptor 2 expression. J Cell Mol Med. 2013;17:1025–1037. 23991686 10.1111/jcmm.12073 PMC3780541

[194] Suzuki S , Ichikawa Y , Nakagawa K , et al. High infiltration of mast cells positive to tryptase predicts worse outcome following resection of colorectal liver metastases. BMC Cancer. 2015;15:840. 26530140 10.1186/s12885-015-1863-z PMC4632336

[195] Granot Z , Jablonska J . Distinct functions of neutrophil in cancer and its regulation. Mediators Inflamm. 2015;2015:1. 10.1155/2015/701067 PMC4663337 26648665

[196] Peddareddigari VG , Wang D , Dubois RN . The tumor microenvironment in colorectal carcinogenesis. Cancer Microenviron. 2010;3:149–166. 21209781 10.1007/s12307-010-0038-3 PMC2990487

[197] Deschoolmeester V , Baay M , Van Marck E , et al. Tumor infiltrating lymphocytes: an intriguing player in the survival of colorectal cancer patients. BMC Immunol. 2010;11:19. 20385003 10.1186/1471-2172-11-19 PMC2864219

[198] Sandel MH , Speetjens FM , Menon AG , et al. Natural killer cells infiltrating colorectal cancer and MHC class I expression. Mol Immunol. 2005;42:541–546. 15607811 10.1016/j.molimm.2004.07.039

[199] Sconocchia G , Eppenberger S , Spagnoli GC , et al. NK cells and T cells cooperate during the clinical course of colorectal cancer. Oncoimmunology. 2014;3:e952197. 25610741 10.4161/21624011.2014.952197 PMC4292408

[200] Dudley AC . Tumor endothelial cells. Cold Spring Harb Perspect Med. 2012;2:a006536. 22393533 10.1101/cshperspect.a006536 PMC3282494

[201] Ribeiro AL , Okamoto OK . Combined effects of pericytes in the tumor microenvironment. Stem Cells Int. 2015;2015:1. 10.1155/2015/868475 PMC4427118 26000022

[202] Raza A , Franklin MJ , Dudek AZ . Pericytes and vessel maturation during tumor angiogenesis and metastasis. Am J Hematol. 2010;85:593–598. 20540157 10.1002/ajh.21745

[203] Chan TE , Stumpf MPH , Babtie AC . Gene regulatory network inference from single‐cell data using multivariate information measures. Cell Syst. 2017;5:251–267.e3.e253. 28957658 10.1016/j.cels.2017.08.014 PMC5624513

[204] Armingol E , Officer A , Harismendy O , Lewis NE . Deciphering cell‐cell interactions and communication from gene expression. Nat Rev Genet. 2020. 10.1038/s41576-020-00292-x PMC7649713 33168968

